# The double-edged role of copper in the fate of amyloid beta in the presence of anti-oxidants†
†Electronic supplementary information (ESI) available. See DOI: 10.1039/c7sc01787a



**DOI:** 10.1039/c7sc01787a

**Published:** 2017-06-22

**Authors:** Jing Yang, Xueli Zhang, Yiying Zhu, Emily Lenczowski, Yanli Tian, Jian Yang, Can Zhang, Markus Hardt, Chunhua Qiao, Rudolph E. Tanzi, Anna Moore, Hui Ye, Chongzhao Ran

**Affiliations:** a Molecular Imaging Laboratory , MGH/MIT/HMS Athinoula A. Martinos Center for Biomedical Imaging , Department of Radiology , Massachusetts General Hospital , Harvard Medical School , Room 2301, Building 149, Charlestown , Boston , Massachusetts 02129 , USA . Email: cran@nmr.mgh.harvard.edu; b College of Pharmaceutical Sciences , Soochow University , Suzhou , 215006 , China; c Center for Drug Discovery , School of Pharmacy , China Pharmaceutical University , Nanjing , 210009 , China; d Department of Applied Oral Sciences , The Forsyth Institute , Cambridge , MA 02142 , USA; e Department of Biology , Loyola University Chicago , Chicago , IL 60660 , USA . Email: hye1@luc.edu; f Department of Parasitology , Zhongshan School of Medicine , Sun Yat-Sen University , Guangzhou , 510080 , China; g Alzheimer’s Disease Research Unit , Department of Neurology , Massachusetts General Hospital , Building 114 , Charlestown , Massachusetts 02129 , USA

## Abstract

The biological fate of amyloid beta (Aβ) species is a fundamental question in Alzheimer’s disease (AD) pathogenesis. The competition between clearance and aggregation of Aβs is critical for the onset of AD. Copper has been widely considered to be an inducer of harmful crosslinking of Aβs, and an important triggering factor for the onset of AD. In this report, however, we present data to show that copper can also be an inducer of Aβ degradation in the presence of a large excess of well-known intrinsic (such as dopamine) or extrinsic (such as vitamin C) anti-oxidants. The degraded fragments were identified using SDS-Page gels, and validated *via* nanoLC-MS/MS. A tentative mechanism for the degradation was proposed and validated with model peptides. In addition, we performed electrophysiological analysis to investigate the synaptic functions in brain slices, and found that in the presence of a significant excess of vitamin C, Cu(ii) could prevent an Aβ-induced deficit in synaptic transmission in the hippocampus. Collectively, our evidence strongly indicated that a proper combination of copper and anti-oxidants might have a positive effect on the prevention of AD. This double-edged function of copper in AD has been largely overlooked in the past. We believe that our report is very important for fully understanding the function of copper in AD pathology.

## Abstract 2

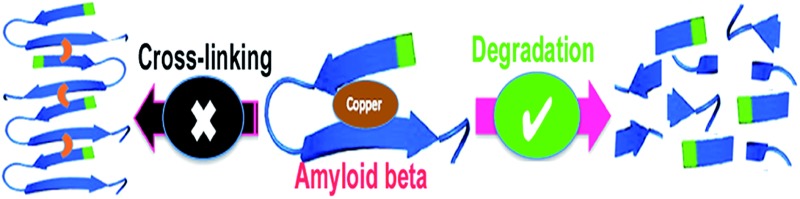
The cleavage of amyloid beta induced by copper(ii) in the presence of anti-oxidants is discussed.

## Introduction

To date, drug development for Alzheimer’s disease (AD) has been largely unsuccessful.^1–6^ However, emerging evidence suggests that alternative approaches, such as adapting to a healthy lifestyle, can significantly improve or maintain cognitive function in at-risk elderly people.^7–10^ Lifestyle adaptation, including a healthy diet, regular physical exercise and cognitive training, can lead to a significant increase in production of intrinsic anti-oxidants such as dopamine and an increase of the uptake of extrinsic anti-oxidants such as vitamin C (Vc). In this report, we provide unexpected *in vitro* evidence that a significant excess of anti-oxidants such as Vc and dopamine can facilitate copper-induced degradation of Aβs. Our results may be relevant to the beneficial effects of a healthy lifestyle on AD prevention and treatment.

Originally, Cu(ii) ion’s effects on Aβ have been considered to be the cause of the harmful cross-linking of Aβs, which significantly contributes to the development of Alzheimer’s disease.^11–14^ However, we recently accidentally discovered that Cu(ii) could also induce Aβ degradation in the presence of extrinsic anti-oxidants such as Vc and endogenous anti-oxidants such as dopamine. It is believed that the cross-linking of Aβ induced by Cu(ii) originates from an oxidative reaction with Aβs. Atwood *et al.* reported that Cu(ii) could coordinate with Histidine 6, 13, and 14 (H6, 13, and 14) of Aβ peptides, and could be further reduced by Vc to initialize the oxidative cross-linking of tyrosine (Y10) of Aβs.^15^ Cross-linking and degradation of the proteins are the two primary outcomes of an oxidative reaction of proteins/peptides.^16–25^ In the case of Aβ, the cross-linking of Aβ by oxidative reactions has been well recognized,^11,12,26,27^ however oxidative degradation of Aβ, to the best our knowledge, has not been intensively investigated and has been overlooked in the past decades.

In the course of our investigation, we used a fluorescent dye-conjugated Aβ (FAM-Aβ42) for preparation of the cross-linking products with Cu(ii) and Vc. Unexpectedly, two fast migrating bands were detected on the SDS-PAGE gels. Their molecular weights were less than 4KD, indicating that they were degraded fragments of Aβs. To investigate the oxidative degradation of Aβs, we first used nanoLC-MS/MS to identify the degraded fragments from native Aβ42 and FAM-Aβ42, and then we proposed a possible degradation mechanism. To further elucidate the mechanism, a peptide fragment was used to mimic the degradation reactions. Following these studies, we used FAM-Aβ42 as a model peptide to examine the effects of different metal ions including Fe(iii), Fe(ii), Cu(ii) and Zn(ii), different reductants including well-known extrinsic compounds such as Vc, curcumin, resveratrol and vitamin E (Ve), and intrinsic compounds such as norepinephrine (NE) and dopamine (DOPA) on the degradation of Aβ. Moreover, we showed that the combination of an anti-oxidant and an anti-aggregating drug could slightly increase the fraction of the degradation products. We also investigated whether the combination of Cu(ii) and anti-oxidants could provide neuronal protection benefits. Remarkably, field excitatory postsynaptic potential (fEPSP) recording on mouse brain slices indicated that the combination of Cu(ii) and a significant excess of Vc could prevent synaptic impairment induced by Aβs.

## Results

### 1. Discovery of the degradation of FAM-Aβ42 by Cu(ii) and Vc

**Fig. 1:**
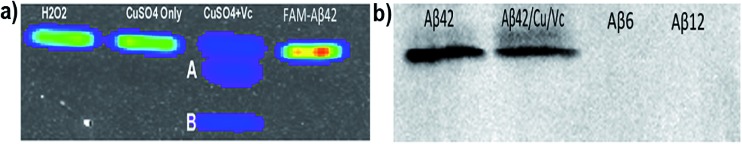
Degradation of Aβ induced by Cu(ii) and Vc. (a) SDS-PAGE of FAM-Aβ42 with H_2_O_2_, Cu(ii), and Cu(ii)/Vc (1 : 10). Two fast migrating bands A and B can be clearly seen. (b) Western blotting with 6E10 antibody for Aβ42, Aβ42/Cu(ii)/Vc (1 : 10), Aβ6 and Aβ12. Aβ6 and Aβ12 cannot be visualized with the antibody.

The combination of Cu(ii) and Vc has been used to investigate the crosslinking of Aβ.^15^ In the course of screening crosslinking inhibitors, we incubated FAM-Aβ42 with copper sulfate and Vc for 24 hours, and the mixture was then subjected to SDS-PAGE gel electrophoresis. Since FAM is a fluorescent dye, we imaged the gel directly on an imaging system. Surprisingly, we observed two fluorescent bands (bands A and B in Fig. 1a) that migrated faster than the monomeric bands on the gel, suggesting that their molecular weights are less than 4KD, and thus also strongly suggesting that they represent degraded segments of FAM-Aβ42 (Fig. 1a). Control experiments with Cu(ii) only were conducted, and no degradation products were observed (Fig. 1a). Opazo *et al.* reported the production of H_2_O_2_ upon Cu(ii) binding to Aβ in the presence of Vc.^28^ To examine whether H_2_O_2_ could induce degradation, we incubated FAM-Aβ42 with different concentrations of H_2_O_2_. No apparent cleavage could be detected, indicating that copper was necessary for the cleavage (Fig. 1a).

### 2. Degradation of native human Aβ42

To investigate whether Cu(ii) could induce degradation of native Aβ42 in the presence of anti-oxidants, we incubated Aβ42 with CuSO_4_ and Vc (Aβ42/Cu(ii)/Vc = 1 : 1 : 10) for 24 hours. Since native Aβ42 itself doesn’t have a direct fluorescence readout like FAM-Aβ42, we attempted to visualize the possible degradation products by western blotting. We observed that the density of the monomeric band from the Cu(ii)/Vc group was lower than that from the control group, indicating that both crosslinking and degradation were possible (Fig. 1b). Unfortunately, we failed to visualize any bands faster than the 4KD band with Aβ antibody 6E10, whose specific epitope is Aβ1–16 (Fig. 1b). Similarly, Aβ antibody 2H4, whose epitope is Aβ1–8, also failed to detect the lower molecular weight bands (data not shown). These failures are likely due to the incapability of the antibodies to recognize the degraded fragments. We verified this speculation by western blotting with standard Aβ6 and Aβ12 peptides. No apparent bands were observed with the antibodies (Fig. 1b), confirming that western blotting was not suitable for quantification of the degradation of the native Aβs.

### 3. Identification of the degraded fragments of native Aβ42 by nanoLC-MS/MS

**Fig. 2:**
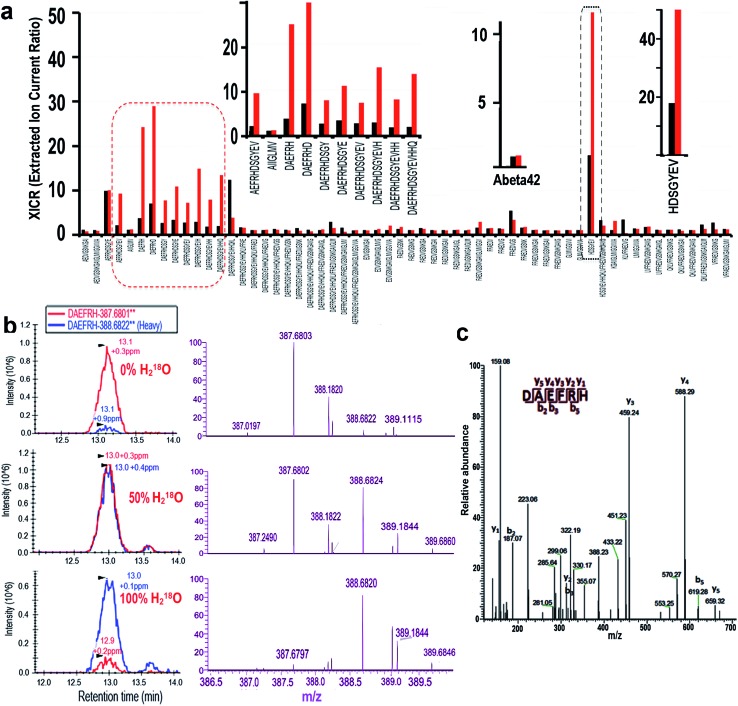
Identification of the degraded fragments of native Aβ42 using nanoLC-MS/MS. (a) Relative intensities of the sequences of interest in 50% (*R*_50%_, black bars) and 100% (*R*_100%_, red bars) of H_2_^18^O buffer. The calculation was based on XICR (*R*_50%_ or *R*_100%_ = XICR_Cu_/XICR_Control_). Left insert: zoomed in image of the red circle; middle insert: XICR of the whole sequence of native Aβ42; right insert: zoomed in image of the black circle. (b) Skyline of nano-LC (left panel, red: normal MS; blue: ^18^O incorporated) and MS (right panel: purple) of a representative degraded fragment, Aβ6 (DAEFRH), in 0% (control), 50% and 100% H_2_^18^O buffer. Note that the MS patterns changed with the increasing H_2_^18^O content. (c) MS/MS of the representative degraded fragment Aβ6 in (b).

To further investigate the observed degradation, we used nanoLC-MS/MS to detect the fragments of Aβ42. In these experiments we used Aβ42 only as the control, and Aβ42/Cu(ii)/Vc as the experimental group. It is well-known that during the degradation of a peptide, H_2_O from the incubation medium participates in the cleavage of the C–N bond *via* incorporating a hydroxyl (OH) into the final carboxylic acid product. In proteomics studies, stable isotope labeling has been widely used to introduce signature mass tags to accurately assign fragments of proteins and peptides.^29–31^ To unambiguously identify the fragments of Aβ42, we took advantage of the stable isotope labeling mass spectrometry method. To this end, we incubated Aβ42, CuSO_4_ and Vc in PBS buffer with 50% and 100% heavy water (H_2_^18^O) respectively to obtain ^18^O labeled degradation products. Our analysis was based on the extracted ion current ratio (XICR) of ^18^O/^16^O (heavy/light) of the sequence of interest. To validate the degraded fragments, we first compared the XICRs of the control and treated groups in the PBS buffer with 50% H_2_^18^O, and plotted a graph by normalizing to the XICR of the control (*R*_50%_ = XICR_Cu_/XICR_Control_) (Fig. 2a, black bars). In principle, the higher the value a sequence has, the higher the probability is that the sequence has ^18^O incorporated, and thus the sequence is more likely to be a cleavage product. From this graph, we identified 60 sequences with *R*_50%_ > 1, which corresponded to the likely degraded fragments (ESI Table 1†). To further validate the candidate sequences, we calculated *R*_100%_ (*R*_100%_ = EICR_Cu_/EICR_Control_) for the sequences obtained from the 100% H_2_^18^O buffer (Fig. 2a, red bars). It is reasonable to assume that, if the sequence is a degraded fragment, *R*_100%_ should be larger than *R*_50%_ (*R*_100%_ > *R*_50%_). With this additional criterion, ten sequences were identified as the degraded fragments (Fig. 2a left and right inserts). To confirm the reliability of our analysis, we analyzed the *R*_100%_ and *R*_50%_ values of the full Aβ42 sequence, which was not totally degraded in the analyzed solution. As expected, the *R*_100%_ and *R*_50%_ values of Aβ42 were very similar (Fig. 2a middle insert), indicating that our analysis was reliable.

The nano-LC, MS, and MS/MS spectra of a representative fragment, DAEFRH, are shown in Fig. 2b and c. Clearly, the MS patterns of 0%, 50%, and 100% H_2_^18^O buffer were shifted, and are consistent with the expectation. The tandem MS/MS pattern also ambiguously identified the sequence of the DAEFRH fragment (Fig. 2c). The nano-LC, MS, and MS/MS spectra of the rest of the fragments are listed in the ESI section (Fig. 1†).

Interestingly, all of the cleaved sites are adjacent to histidines (H6, H13 and H14), whose imidazoles can coordinate with Cu(ii), indicating that the specificity of the degradation was related to histidine. Histidine-specific oxidative degradation of proteins/peptides in the presence of metal ions has been reported,^32^ and our results are consistent with the reported reference.

In addition, it has been reported that copper could oxidize histidine to generate imidazolone;^33–35^ therefore it is necessary to investigate whether fragments containing imidazolone can be validated. Using the same criteria for identifying the non-oxidized fragment, we confirmed that oxidized fragments of Aβ42 could be identified from the collection of nanoLC-MS/MS data sets from 50% and 100% heavy water incubation. The nano-LC, MS, and MS/MS of the oxidized fragments are listed in ESI Fig. 1.†

### 4. Identification of the FAM-Aβ42 fragments

To investigate the origin of the peptide fragments of the two fast migrating bands A and B in Fig. 1a, we used the same protocol as that of nanoLC-MS/MS for native Aβ42. FAM-Aβ6 was first identified (ESI Fig. 2†). For further confirmation, standard FAM-Aβ6 was used for electrophoresis, and the same mobility was confirmed for this fragment as for the fastest migrating band B (ESI Fig. 3a†). Initially, we speculated that FAM-Aβ12 was the fast-migrating band A; however, the mobility of the standard FAM-Aβ12 was not the same as band A (ESI Fig. 3a†). Our data from the native Aβ42 suggested that oxidized fragments were possible. To confirm this we incubated standard FAM-Aβ12 with CuSO_4_ and Vc, and found that the mobility of the new band was consistent with band A, indicating that band A was the oxidized FAM-Aβ12 (ESI Fig. 3b†).

### 5. Comparison of the native gel and SDS-gel for Aβs

To investigate whether the cleavage behaviors of Aβs are different between the native gel and SDS-gel, we performed an electrophoresis experiment with SDS-gel and native gel for FAM-Aβ42. We found no apparent cleavage difference between the two gel images. Similar to the results with the SDS-gel, two fast moving bands (cleaved fragments) could be clearly seen from the native gel imaging (ESI Fig. 3c†). We also investigated the aggregation behavior of Aβs with these two types of gel. We did not observe signals from the aggregated Aβs (high molecular weight > 20 kD) with FAM-Aβ42 from both gels (ESI Fig. 3c†), which is likely due to the high degree of aggregation that could quench the fluorescence signal (high local concentration of FAM). However, we also noticed some differences between the two gels. Two low MW bands of oligomers could be clearly observed with the SDS-gel, but not with the native gel. It is not clear what caused this difference. To avoid the possible quenching effect of FAM dye, we performed western blotting for FAM-Aβ42 with both the SDS-gel and native gel, and found that aggregation did occur (ESI Fig. 3d†). This result is consistent with previous reports.^11–15,36^ In addition, we conducted western blotting for the native Aβ42, and images from both gels showed that Cu(ii)/Vc did induce aggregation of the native Aβs (ESI Fig. 3e†). This is similar to the result from FAM-Aβ42. Taken together, our data indicated that Cu(ii)/Vc could induce both cleavage and aggregation of Aβs. However, in this report, our focus was the cleavage of Aβs, not the aggregation of Aβs.

### 6. Effect of oxygen on the cleavage of Aβ peptides

To investigate whether oxygen is crucial for the cleavage, we performed reactions of FAM-Aβ42 with Cu(ii) under both aerobic and anaerobic conditions with and without anti-oxidants. Anaerobic experiments were performed in an O_2_-free glove-box, and all the solutions were prepared anaerobically by purging with high purity nitrogen gas. As shown in ESI Fig. 4a and b,† the degradation of Aβs under anaerobic conditions was significantly decreased compared to the aerobic conditions, suggesting that oxygen is essential for degradation induced by Cu(ii)/Vc. However, it is difficult to have a completely air-free environment for our experiments, and this could provide an explanation for some cleavage that could still be observed under the anaerobic conditions. For the cleavage induced by Cu(ii)/H_2_O_2_, our data suggested that oxygen was not essential (ESI Fig. 4a and b†).

### 7. Mechanism studies for the cleavage of Aβ peptides

To investigate the possible mechanism of the copper induced cleavage, we first used a short Aβ fragment, YEVHH, as a model peptide, in which Cu(ii) can coordinate with the imidazoles of the two histidines. The fate of the peptide in the presence of CuSO_4_ and Vc was monitored by LC-MS to investigate: (1) whether the model peptide can be cleaved; (2) if cleaved, what were the products; (3) how the concentrations of the starting peptide and newly generated fragments change at different time points. We found that YEVHH could be cleaved by Cu(ii)/Vc, resulting in total degradation after 24 hours incubation (ESI Fig. 5a†). At 3.5 hours incubation, we found two major products, one being YEVHH + 16 Da, corresponding to the oxidized product, and another being YEV, corresponding to the cleaved product. Meanwhile, a dramatic decreasing of the intensity of YEVHH + 16 Da was observed during the period of incubation of 3.5 hours to 24 hours. Nonetheless, the intensity of YEV was significantly increased (ESI Fig. 5a†), indicating that YEVVHH + 16 Da was the intermediate for the cleavage.

Based on the above results, we proposed a tentative mechanism to explain the cleavage (ESI Fig. 5b†). Several reports^37–42^ have indicated that Cu(ii) could coordinate with three imidazoles from the Aβ peptide (H6, H13 and H14) (ESI Fig. 5b†). In the presence of Vc, the Cu(ii) complex could be reduced to a Cu(i) complex, which is ready to accept dioxygen (O_2_) to form a Cu(i)–O_2_ complex.^42–47^ According to previous references, the Cu(i)–O_2_ complex could undergo several steps to produce reactive HO˙ (ESI Fig. 5b†), which could attach to the nearby imidazole to provide an imidazolone (also called 2-oxo-imidazole) intermediate. Our data from the mimic experiments and reports^35,48–50^ supported the existence of imidazolone (ESI Fig. 5a†). Based on the reported mechanism for the oxidative cleavage of peptides,^16,50^ HO˙ could also further attack the α-position of the nearby amide bond to generate a free radical intermediate, in which the amide bond will be cleaved with the assistance of H_2_O (ESI Fig. 5b†).

### 8. Copper/Vc ratio for degradation

**Fig. 3:**
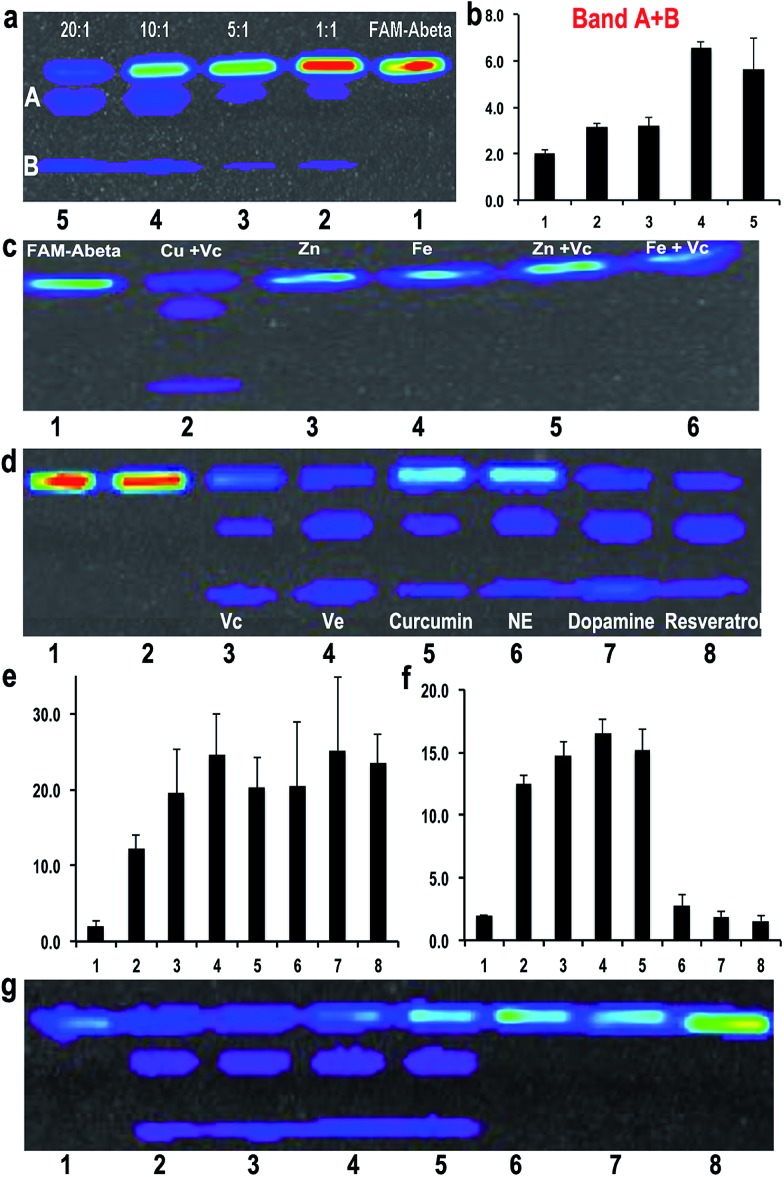
(a) SDS-PAGE of FAM-Aβ42 with different ratios of Cu(ii)/Vc (1 : 1, 1 : 5, 1 : 10 and 1 : 20). (b) Quantitative analysis of the cleaved bands for each condition in (a) (*n* = 3). (c) SDS-PAGE of FAM-Aβ42 with Cu(ii)/Vc, Zn(ii), Fe(iii), Zn(ii)/Vc, and Fe(iii)/Vc. (d) SDS-PAGE of FAM-Aβ42 with CuSO_4_ and different anti-oxidants, including Vc, vitamin E (Ve), curcumin, NE, dopamine, and resrevatrol. FAM-Aβ42 (Lane 1); FAM-Aβ42/Cu(ii) 1 : 1 (Lane 2); FAM-Aβ42/Cu(ii)/anti-oxidants 1 : 1 : 20 (Lanes 3–8). (e) Quantitative analysis of the cleaved bands for each condition in (d) (*n* = 3). (f) Quantitative analysis of the cleaved bands for each condition in (g) (*n* = 3). (g) SDS-PAGE of FAM-Aβ42 with the combination of Cu(ii)/Vc and anti-aggregating drug clioquninol (CQ). FAM-Aβ42 (Lane 1); FAM-Aβ42/Cu(ii)/Vc 1 : 1 : 20 (Lane 2); Lanes 3–8: FAM-Aβ42/Cu(ii)/Vc/CQ = 1 : 1 : 20 : 0.1 (Ln3), 0.5 (Ln4), 1.0 (Ln5), 2.0 (Ln6), 5.0 (Ln7), 10.0 (Ln8).

To investigate the efficiency of the cleavage with the combination of Cu(ii) and Vc, we incubated FAM-Aβ42 with different ratios of Cu(ii)/Vc varying from 1 : 1 to 1 : 20. The results indicated that the ratio was critical for efficient degradation, with a high Vc ratio promoting more efficient cleavage. This strongly suggests that the ratio of Cu(ii) and Vc is the key for determining the fate of Aβ peptides (Fig. 3a and b). For quantification, we used the total ROI signal from bands A and B, and normalized the values to total ROI signals from the same positions of bands A and B in the control FAM-Aβ42 lane. Interestingly, we also noticed that a low ratio/concentration of Cu(ii) could not significantly induce the cleavage of Aβs (ESI Fig. 6a†), which is likely due to the requirement of coordination between Cu(ii) and the Aβ peptide.

### 9. The degradation effect of Zinc(ii), Fe(iii) and Fe(ii)

It is well-known that Zn(ii) and Fe(iii)/Fe(ii) play important roles in triggering AD.^51^ Over-accumulation of these metal ions has been found around the senile plaques. To test whether these metal ions could also lead to degradation of Aβ, we conducted experiments with Zn(ii), Fe(iii), Zn(ii) + Vc, and Fe(iii) + Vc. No obvious degraded products were observed with Zn(ii) or Zn(ii) + Vc, probably due to the incapability of zinc(ii) to induce oxidation under this condition (Fig. 3c). Although it was reported that Fe(iii) could induce protein/peptide cleavage,^23^ no FAM-Aβ42 degraded products were detected with either Fe(iii) or Fe(iii) + Vc (Fig. 3c). In addition, there was no apparent degraded fragment when FAM-Aβ42 was incubated with Fe(ii) with and without Vc under both aerobic and anaerobic conditions (ESI Fig. 6b†). This data may indicate that the degradation of FAM-Aβ is Cu(ii) specific.

### 10. Degradation of Aβs with other well-known anti-oxidants

To investigate whether other well-known anti-oxidants are capable of promoting the degradation of FAM-Aβ42, we tested extrinsic vitamin E (Ve), curcumin, resveratrol, endogenous dopamine and norepinephrine as reducing reagents. We found that all of these anti-oxidants could lead to the degradation of FAM-Aβ42 in the presence of Cu(ii) (Fig. 3d and e). Remarkably, the intrinsic anti-oxidants dopamine and norepinephrine are tightly related to neuron activity, with dopamine being one of the important molecules released from synapse activation. A low dopamine level has been linked to AD.^52^ In addition, we also compared the effect of curcumin to its analogue CRANAD-5, which doesn’t contain a phenolic group as a reducing compound^53^ (ESI Fig. 7†). No degradation products were detected with CRANAD-5, indicating that the cleavage induced by curcumin originated from the oxidative reaction.

### 11. A combination of anti-oxidant and anti-aggregating drugs increases the degree of degradation

The above results clearly indicate that Cu(ii) plays a double-edged sword role in the fate of Aβ peptides. In principle, aggregation and degradation are two competitive paths. Therefore, in the presence of a compound that can reduce the aggregation of Aβs, the chance of Aβs to undergo Cu(ii)-induced degradation would increase. To investigate this hypothesis, we selected clioquinol (CQ), which is an antifungal and anti-protozoal drug, and also has been clinically tested for AD. Reportedly, CQ can reduce the aggregation of Aβs *in vitro* and significantly lower the loading of Aβ plaques in transgenic mice.^54,55^ We tested the combination of CQ/Cu(ii)/Vc with various concentrations of CQ (the molar ratio of CQ/Cu(ii): 0.1–10).

Indeed, we found that low concentrations of CQ could promote the degradation of FAM-Aβ42 (Fig. 3f and g). However, high concentrations of CQ prevent the degradation, probably due to the coordination of CQ with Cu(ii). We also tested *scyllo*-inositol (AZD-103), a natural plant sugar currently in clinical trials for AD therapy, as the anti-aggregating compound.^56,57^ However, no significant increase in the degradation was observed (data not shown).

### 12. Degradation of insoluble Aβ40 aggregates and oligomers

**Fig. 4:**
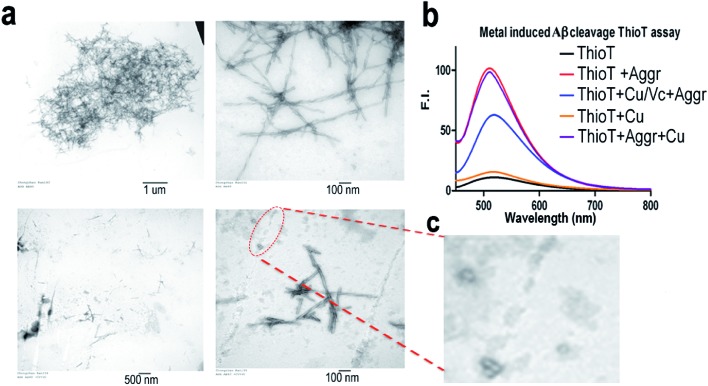
The degradation of the native Aβ40 aggregates induced by Cu(ii)/Vc. (a) TEM image of the Aβ40 aggregates before degradation (top) and after degradation (bottom). Right panel: high magnificent image. (b) Thioflavin T fluorescence testing with the Aβ40 aggregates before degradation (red) and after degradation (blue). (c) Zoomed in image of the debris of a degraded fibril.

To investigate whether the combination of Cu(ii)/Vc can degrade the aggregated Aβs, we incubated Aβ40 aggregates with CuSO_4_ and Vc for 72 hours. TEM images show that the aggregates/fibrils could be degraded forming much shorter fibrils (Fig. 4a and c). The debris from degraded fibrils can be clearly seen in Fig. 4c. When the incubated solutions were tested with thioflavin T, we observed a significant decrease of fluorescence response in the presence of CuSO_4_ and Vc (Fig. 4b). A control experiment showed that CuSO_4_ had no significant effect on the fluorescence of ThioT. These results suggested that the aggregated Aβs could be degraded by the combination of Cu(ii) and Vc.

Mounting evidence suggests that Aβ oligomers are more toxic than insoluble Aβs.^58–61^ To investigate whether the Aβ oligomers could be degraded by Cu(ii)/Vc, we first used TEM to characterize the Aβ oligomers. TEM showed that the size of the Aβ oligomers is around 100 nm. We then compared the sizes of the Aβ oligomers with and without Cu(ii)/Vc treatment, and found that the size of the treated group was significantly smaller than that of the control group (ESI Fig. 8†), indicating that Cu(ii)/Vc is able to degrade the Aβ oligomers. However, in this report we did not intend to compare the degradation speeds of different Aβ species.

### 13. Proliferation studies with neuronal cells

To investigate whether the combination of Cu(ii) and Vc can reduce the neurotoxicity of Aβs, we conducted a proliferation assay with SH-SY5Y neuronal cells treated with (1) Aβ42 (20 μM), (2) Aβ42 and Vc (1 : 10), (3) Aβ42 and Cu(ii) (1 : 1), and (4) Aβ42, Cu(ii) and Vc (1 : 1 : 10) for 4 hours and 24 hours. Our data indicted that Aβ42 could indeed apparently reduce the cell survival rate while Vc alone could not rescue the neurotoxicity of Aβ42. Among the treatments, the lowest cell survival rate (64%) was in the presence of Aβ42 and Cu(ii), which could be partially reversed with the addition of a large excess of Vc (80%) (ESI Fig. 9†).

### 14. Cu(ii)/Vc combination rescues the Aβ impaired synaptic transmission (fEPSP) in the hippocampus

**Fig. 5:**
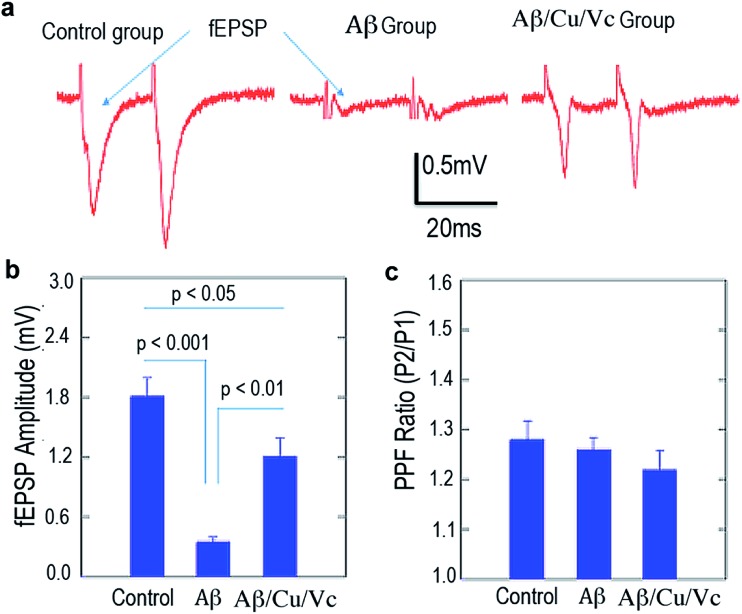
(a) fEPSP recording of mouse brain slices treated with the vehicle (left), Aβ42 (middle), and Aβ42/Cu(ii)/Vc (right). (b) Quantification of the fEPSP in (a). (c) Quantification of the PPF ratio of the vehicle, Aβ42, and Aβ42/Cu(ii)/Vc.

In AD, dementia severity correlates strongly with decreased synapse density in the hippocampus and cortex. To characterize the detrimental effects of Aβs in synaptic transmission in the hippocampal CA1 area, we stimulated the Schaffer collateral pathway once every 15 s and recorded extracellularly from the stratum radiatum. The fEPSP, which represents the summarized synaptic events from a large population of terminals, was measured as the negative deflection of the voltage trace from the baseline (Fig. 5a and b). We quantified the maximal fEPSPs in the control, Aβ, and Aβ/Cu(ii)/Vc groups. Repeated measures by ANOVA of the three groups showed significant differences among these groups (*p* < 0.0001). Bonferroni multiple comparisons test in different group pairs showed that the maximal fEPSP amplitudes were significantly smaller in the Aβ-treated slices than in the control groups (control: 1.81 ± 0.18 m; Aβ: 0.35 ± 0.05 mV, *p* < 0.001). Treatment with Cu(ii)/Vc prevented the Aβ-induced impairment in synaptic transmission. The maximal fEPSP amplitude (1.21 ± 0.18 mV) in the Aβ/Cu(ii)/Vc group is significantly larger than that in the Aβ group (*p* < 0.01).

To characterize synaptic plasticity in the C1 area, we delivered paired pulses (50 ms interval) to the presynaptic terminals, and recorded the ratio of the fEPSPs (PPF) triggered by the 2^nd^ pulse to the 1^st^ pulse. Repeated measures by ANOVA of the three groups showed no differences among these groups (*p* = 0.46). Bonferroni multiple comparisons test in different group pairs showed that the PPF ratios were not different among the three groups (Fig. 5c), indicating that the short-term plasticity was not impaired.

## Discussion

Metal ions play numerous roles in various biological processes. Their proper balance is crucial for the normal function of a living being. Copper is an essential metal ion for numerous metalloproteins, such as ceruloplasmin and cytochrome oxidase.^62^ The deficiency of copper can lead to various pathologies such as Menkes disease and Wilson disease.^63^ On the other hand, over-accumulation of copper can reportedly contribute to AD, schizophrenia, and obsessive-compulsive disorder.^51,63^ For AD pathology, copper has been considered as an important trigger in the harmful crosslinking of Aβs. In this report, we demonstrated that copper could play a double-edged sword role in the fate of Aβs. Our results are not contradictory but complementary to previous studies, and are important for the complete understanding of the function of copper in AD pathology.

The degradation of Aβs by copper has largely been overlooked in the past, likely due to the inability of the available Aβ antibodies to recognize small degraded fragments using western blotting. Harnessing the capability of nano LC-MS/MS, we clearly showed that Aβs could be degraded by copper in the presence of anti-oxidants. Although the cleavage of Aβ model peptides has been reported, most of the degraded products identified in our studies differ from previously reported fragments, except for the AEFRHDSGYEV fragment, which shares the same cleavage site D1 (aspartate) at the N-terminal of the Aβ28 model peptide as identified by Cassagnes *et al.* ^64^ We also identified cleavage sites that are different from Drew’s report, which defines E3 as the cleavage site^65^ using the Aβ16 model peptide. The difference between our results and those by Cassagnes *et al.* and Drew *et al.* could probably be ascribed to the fact that we used more comprehensive methods for the analysis of the degraded fragments. In our studies we not only used LC-MS, but also ran SDS-PAGE to visualize the “visible” FAM-Aβ fragments, which allowed us to ambiguously identify the degraded products. Moreover, instead of Aβ16 and Aβ28 that were used as the model peptides in Cassagnes and Drew’s reports,^64,65^ we used the full Aβ42 sequence, which more accurately reflects the degradation of Aβ42. Wu *et al.* designed a series of copper complexes that could promote the cleavage of Aβs, and the cleavage sites were similar to our results.^66^ In addition, our mechanism studies with the model peptides support the cleavage sites identified in this report.

While this manuscript was under review, Derrick *et al.* reported that Aβ peptides could be cleaved by complexes of metal ions and tetra-*N*-methylated cyclam (TMC) *via* an intermolecular interaction mechanism.^67^ Data suggested that hydrolysis of the amide bond of Aβs caused the cleavage, likely due to the complex [Co(ii)(TMC)(H_2_O)]^2+^ leading to a local increase in the concentration of hydroxide nucleophiles near the peptide bonds.^67^ Interestingly, this cleavage mechanism is quite different from our proposed intramolecular metal binding mechanism. Our data suggested that the cleavage was likely due to the formation of an Aβ-Cu(i)–O_2_ intermediate and free radicals that consequently cause the cleavage of the nearest amide bond(s) of the Aβs. Although the mechanisms were different, the cleavage efficiencies of both mechanisms were comparable under pH 7.4 conditions (about 60% of Aβs are cleaved, calculated from Fig. 2e in ref. 67).

Iron, copper and zinc are the metal ions that have been intensively investigated for AD pathology. Interestingly, among the three metal ions, only copper can induce degradation. It is not clear why Fe(iii) and Fe(ii) could not initialize the degradation. For Fe(iii), it is likely due to its weak coordination with Aβ peptides.^68,69^ Fe(ii) could not be reduced by Vc, and this probably could provide an explanation for our results.^68,69^ Zn(ii) is not able to induce degradation, which is likely due to its incapability to participate in redox reactions.

Our results showed that Aβ degradation was dependent on Cu(ii) and Vc concentration. With the increase of the Vc concentration, the degree of degradation was also increased, and a low concentration of Cu(ii) could not significantly induce the degradation. We also found that Aβ degradation could be promoted not only by extrinsic anti-oxidants such as Vc, Ve, resveratrol, and curcumin, but could also be enhanced by intrinsic anti-oxidants such as dopamine and norepinephrine. A low dopamine level has been linked to AD.^52^ We believe that these results support the beneficial effects of a healthy lifestyle on AD management. A healthy diet can increase the uptake of vitamins such as Vc, Ve, and reservatrol, and physical exercise can increase the release of dopamine and norepinephrine.

Our data indicated that copper could play a double-edged sword role in the fate of Aβs. Copper-induced harmful crosslinking/aggregation and degradation are competitive paths. If the aggregation path can be suppressed, more Aβs will be degraded. Our data support this hypothesis. We found that the combination of anti-aggregation agent and Vc could promote more degradation. Our results suggested that a formulation of CQ or PBT-2 (ref. 54 and 55) with reservatrol or other BBB penetrating anti-oxidants could be beneficial for AD treatment.

Our preliminary data are supported by cell proliferation studies and fEPSP studies with brain slices. Our *in vitro* Aβ treatment caused significant impairment in the basic synaptic transmission, as revealed by the decreased fEPSP magnitude. Synaptic transmission impairment has been observed in several AD animal models.^70–73^ The mechanism is largely unclear, but could be attributed to the Aβ-induced abnormal handling of calcium influx,^74–76^ oxidative stress,^77^ and abnormal activation of ion channels on the presynaptic cells.^73^ Interestingly, we did not observe quantitative changes in the PPF ratio in Aβ challenged slices. It has been reported that in the early stages of AD, there is no PPF ratio change in AD transgenetic CRND 8 animals.^70^ We speculated that the *in vitro* Aβ challenge implored in our experiments could only cause mild changes in the electrophysiological profiles, including fEPSP, but the short term plasticity is intact. Our results indicated that copper with a large excess of Vc could rescue the Aβ-induced deficit in synaptic transmission. It would be interesting to test whether the rescue effects of Cu(ii)/Vc could be attributed to the relief of oxidative stress or prevention of abnormal ion channel functionality.

In conclusion, we would like to reiterate the following points. First, our results do not contradict previous data, which showed the harmful effects of copper on AD pathology. Instead, our studies provide the full picture of copper function in the course of AD pathology. Second, our data indicated that a large excess of anti-oxidant is necessary for achieving more degradation. Third, our studies were conducted *in vitro* and in mouse brain slices, and the actual *in vivo* effect of the combination of copper and a large excess of anti-oxidant is yet to be confirmed. Therefore, more studies are warranted for the investigation of the *in vivo* beneficial effects of the combination.

## Materials and methods

Materials, methods, and chemical syntheses are described in the ESI.† All animal experiments were approved by the Institutional Animal Use and Care Committee at Massachusetts General Hospital and Loyola University Chicago, and all experiments were performed in compliance with the relevant laws and institutional guidelines.
