# Supplementary material for: The double-edged role of copper in the fate of amyloid beta in the presence of anti-oxidants
Source: Chem Sci. 2017 Jun 22;8(9):6155–64. doi: 10.1039/c7sc01787a (PMC5627602; doi:10.1039/c7sc01787a)
Supplement: Supplementary file 1 [file SC-008-C7SC01787A-s001.pdf]

Electronic Supplementary Material (ESI) for Chemical Science.
This journal is © The Royal Society of Chemistry 2017

Index for supplemental information

1. Material and Methods ……………………………………………………………Page 2-7

2. Supplemental Table

SI Table 1……………………………………………………………………………Page 8-9

3. Supplemental Figures

SI Fig.1……………………………………………………………………………Page 10-22
SI Fig.2……………………………………………………………………………Page 23-26
SI Fig.3……………………………………………………………………………Page 26
SI Fig.4……………………………………………………………………………Page 26
SI Fig.5……………………………………………………………………………Page 27
SI Fig.6……………………………………………………………………………Page 28
SI Fig.7……………………………………………………………………………Page 28
SI Fig.8……………………………………………………………………………Page 28
SI Fig.9……………………………………………………………………………Page 29

Material and methods

General procedure

Reagents used were purchased from Sigma-Aldrich and used without further purification. The pH of the PBS buffer was 7.4. Synthetic Aβ peptides (1−40/42) were purchased from rPeptide (Bogart, GA, 30622). FAM-Aβ42 was purchased from American Peptide Company (Vista, CA, 92081). Other peptides were synthesized by Genscript (Piscataway Township, NJ 08854). Aggregates for in vitro studies were generated by the slow stirring of Aβ40 in PBS buffer (0.1% ammonia hydroxyl) for 3 days at room temperature. Fluorescence measurements were carried out using an F-4500 fluorescence spectrophotometer (Hitachi). All animal experiments were approved by the Institutional Animal Use and Care Committee at Massachusetts General Hospital and Loyola University Chicago. A total of C57BL/6J mice (n=10, 2-3 month of age, Charles River Laboratories, Wilmington, MA) were used in this study. Mice were kept in the Loyola University Chicago Animal Facility under continuous care by facility technicians.

Gel electrophoresis and Western blotting:

1) Sample preparation: All the samples used for SDS-PAGE and Western blot were prepared using the same procedure as described below. A 10 μL HFIP (hexafluoroisopropanol) solution (25 μM) of FAM-Aβ42 or native Aβ42 was added to a 1.5 mL LoBind eppendorf tube. After evaporating the organic solvent under vacuum, a 4 μL DMSO was added to the tube, followed by the addition of 8 μL of Vitamin C solution in PBS (312.5 μM) and 8 μL of copper sulfate solution in PBS (31.25 μM). The resulting mixture was incubated at 37°C for 24 hours, and was then subjected to gel electrophoresis. For Cu/Vc ratio study, 8 μL of Vitamin C PBS solution of different concentrations (31.25, 156, 312.5, 625 μM) was added to obtain Cu/Vc ratio equals 1:1, 1:5, 1:10 and 1:20. For dose dependency study, 4 μL of AZD-103 or Cliquinol solution in DMSO of different concentrations (6.25, 31.25, 62.5, 125, 312.5, 625 μM) was added to obtain FAM-Aβ42/AZD-103 or Cliquinol ratios equal 1:0.1, 1:0.5, 1:1, 1:2, 1:5 and 1:10.

For the anaerobic study, all of the solvents that were used to prepare the samples were purged with high purity nitrogen gas, and the preparation and incubation of the samples were performed in an O_2-free glove-box.

2) Gel electrophoresis and blotting: Samples were separated on 4–20% gradient Tris-glycine mini

gels (Invitrogen). For FAM-Aβ42, the images were acquired on IVIS®Spectrum (Perkin Elmer) with excitation = 465nm, and emission = 520nm. For native Aβ42, the gel was transferred onto a PVDF membrane in a cooled transfer buffer, and the membrane was blocked at room temperature for 2 hours with 5% none fat milk. After blocking, the membrane was incubated in a solution of anti-Aβ primary antibody 6E10 (1:2000 dilution, Covance, Dedham, MA) at 4°C overnight. After washing with TBST buffer, the membrane was incubated with the secondary antibody for 1 hour at room temperature. Western Pico Chemiluminescent Substrate (Thermo scientific) was used to visualize the bands. The images were acquired with IVIS®Spectrum using bioluminescence imaging setting. SeeBlue®plus2 (Invitrogen) (4-250KD) was used as a molecular weight marker. Native gel (4-20%) electrophoresis was performed in Tri-Glycine running buffer (without SDS) and blotting was performed with the same protocol as SDS gel blotting.

Nano LC-MS/MS for identifying degraded fragments of Aβ42:

1) Preparation of Aβ42 samples: For the control group: A 5 μL HFIP solution (250 μM) of FAM-Aβ42 or native Aβ42 was added to a 1.5 mL LoBind eppendorf tube. After evaporating the organic solvent under vacuum, 10 μL DMSO was added to the tube, followed by the addition of 40 μL PBS (0.01M, pH=7.4, in H_2O or 50% $H_2{}^{18}O$ or 100% $H_2{}^{18}O$). For the treatment group: A 5 μL HFIP (hexafluoroisopropanol) solution (250 μM) of FAM-Aβ42 or native Aβ42 was added to a 1.5 mL LoBind eppendorf tube. After evaporating the organic solvent under vacuum, 10 μL DMSO was added to the tube, followed by the addition of 20 μL $CuSO_4$ solution (62.5 μM in PBS) and 20 μL Vc solution (1.25 mM in PBS). For the sample containing 50% $H_2{}^{18}O$, 20 μL PBS (solved in $H_2{}^{18}O$) was added to the tube followed by the addition of 10 μL $CuSO_4$ solution (125 μM in PBS) and 20 μL Vc solution (2.5 mM in PBS). For the sample containing 100% H_2O^{18}, 38 μL PBS (solved in H_2O^{18}) was added to the tube, followed by the addition of 1 μL $CuSO_4$ solution (1.25 mM in PBS) and 1 μL Vc solution (25 mM in PBS). All the prepared samples were incubated at 37°C for 24 hours, and were then subjected to nano LC-MS/MS.

2) NanoLC-MS/MS procedure: Aβ42 samples were first purified by Pierce® C18 pipette tips (100 μl, Thermo Scientific). The elute was concentrated by CentriVap and reconstituted in 0.1% formic acid (FA). Peptides were then analyzed by an Orbitrap Fusion™ Tribrid™ mass spectrometer (Thermo Scientific) coupled to an EASY-nLC 1000 Liquid Chromatograph (Thermo Scientific). Samples were loaded onto a trap column (Acclaim PepMap100, 100 μm*2 cm, C18, 5 μm, 100 Å)

(Dionex) and separated by an **EASY-Spray**^{TM} analytical column (Acclaim PepMap, 75 µm *15 cm, C18, 3 µm, 100 Å). Solvent A was 0.1 % FA in water and solvent B was 0.1% FA in CH3CN. Peptides were separated using a 2% to 32% B gradient over 30 min and continued to 72 % B over 3 min at a constant flow rate of 300 nl/min. Full MS scans were obtained with a range of m/z 350 to 2000 at a mass resolution of 120,000 (m/z 200), with an AGC target value of 3E5 and maximum injection time at 250 ms. Twenty most abundant ions were selected, and isolated within a window of 2.5 m/z and fragmented by high-energy collision-induced dissociation (HCD) at a normalized collision energy of 30. MS/MS spectra were acquired at a mass resolution of 15,000 (m/z 200) in Orbitrap with an AGC target value of 2E4 and maximum injection time at 47 ms. Peptides with charge states from 1 to 8 were included. Dynamic exclusion was set to 30 seconds. Data files were searched by Mascot (v2.5) against a self-built database containing the sequence of Aβ42. Digestion Enzyme was specified as none. A precursor ion tolerance of 10 ppm and a product ion tolerance at 0.05 Da were allowed. Variable modifications were set at FAM addition (+358.0477) on N-terminus of peptides as well as oxidation of Histidine. MS/MS spectra, which have Mascot scores higher than 20 and are also within a 0.05 identity significance threshold were selected for O^{18} quantification study. The spectra of main peptides were further inspected manually to validate identification. Peak areas of extracted ion chromatograms for targeted peptides were generated using Skyline (v3.1).

Degradation mechanism investigation via LC-MS: A 30 µL of YEVHH stock solution (2.5 mM in dd water) was added to a 1.5 mL LoBind eppendorf tube. Then 30 uL of $CuSO_4$ solution (2.5 mM in PBS) was added, followed by the addition of 30 µL Vc solution (25 mM in PBS). After incubated at 37°C for 0, 3.5, 7.5, 24 hours, the prepared sample was then subjected to LC-MS, which was performed on an Agilent 1100 Series apparatus with an LC/MSD trap and Daly conversion dynode detector with UV detection at 220, 254, and 280 nm. HPLC column was an Alltima C18 (Grace), 10 µm, 250 × 4.6 mm, and mobile phases are consisted of A (water with 0.1% TFA) and B (MeCN with 0.1% TFA). 20 µL of the sample was injected, and eluted with a gradient of 5 % B to 95 % B over 10 min with a rate of 1.0 mL/min. Detection was in a positive ion mode.

Thioflavin T assay: For the control group, 2.5 µL Aβ40 aggregates (25 µM) was added to 22.5

μL PBS. For the treatment group, 2.5 μL Aβ40 aggregates (25 μM) was added to $CuSO_4$ solution (2.5 μM, 22.5 μL) or solution mixed with $CuSO_4$ (6.25 μM, 10 μL), Vc (62.5 μM, 10 μL) and PBS (2.5 μL). The resulting mixtures were incubated at 37°C for 72 hours. 10 μL of each of the above solutions was diluted to 1.0 mL PBS, and followed by the addition of 10 μL of Thioflavin T (2.5 μM in PBS). The fluorescence spectra were recorded with excitation = 430 nm, and emission = 450 – 800 nm.

TEM Imaging:

1) Sample preparation: For Aβ aggregates, 2.5 μL Aβ40 aggregates (25 μM) was added to 2.5 uL PBS, followed by the addition of $CuSO_4$ 10 μL (6.25 μM) and Vc 10 μL (62.5 μM). The resulting mixtures were incubated at 37°C for 72 hours. A solution of Aβ40 aggregates (2.5 μM) with PBS was prepared as the control. For Aβ oligomers, 20 μL Aβ42 oligomers (2.5 μM) was added to solution mixed with $CuSO_4$ (5.0 μM, 10 μL) and Vc (50 μM, 10 μL) in PBS. The resulting mixtures were incubated at 37°C for 24 hours. A solution of Aβ42 oligomers only (1.25 μM) in PBS was used as the control.

2) TEM imaging procedure: 5 μL of the prepared sample was dropped to a Formvar coated TEM grid, followed by the addition of 2 μL of a PTA contrast solution to the grid. After one minute, the liquid on the grid was carefully dried with a corner of filter paper, and the resulting grid was further dried in the air for 2–5 minute. The TEM images were obtained with a JEOL 1011 electron microscope.

MTT proliferation assay: SH-SY5Y neuronal cell lines were seeded at 5×10^3 cells/well in 96-well microtiter plates. After 24h, cells were exposed to Aβ42 (10 μM); Aβ42 and Vc (1:10); Aβ42 and $CuSO_4$ (1:1); Aβ42, $CuSO_4$ and Vc (1:1:10) for 4 hours and 24 hours. Then cell survival was determined by the addition of an MTT solution (20 μL of 5 mg/mL MTT in PBS). After 6 h, the medium was removed by aspiration. The cells were dissolved in 150 mL DMSO, and optical absorbance was measured at 570 nm on a SpectraMax M2 Microplate Reader (Molecular Devices). Survival ratios were expressed in percentages with respect to untreated cells.

fEPSP recording for investigating the effects of Aβ42/Cu(II) and Aβ42/Cu(II)/Vc on synaptic transmission: *1) Tissue Preparation:* Mice were anaesthetized with isoflurane in a

sealed chamber. Transcardial perfusion was performed with ice-cold oxygenated (95% O_2 +5% CO_2) sucrose-based artificial cerebrospinal fluid (ACSF). The animals were then decapitated and the brain was quickly removed, hemisected, and placed into ice-cold high sucrose ACSF for ~3 min. The high sucrose dissection solution contained (mM): 210 sucrose, 26 $NaHCO_3$, 2.5 KCl, 1 $CaCl_2$, 4 $MgCl_2$, 1.25 NaH2PO$_4$, and 10 d-glucose (pH 7.4, 295 mOsmol), and was saturated with 95% O_2 +5% CO_2. This solution has been shown to enhance tissue viability, permitting long-term recordings of synaptically evoked currents. The cerebellum was removed and the brain was bisected along the mid-sagittal line. The superior cortex was removed and the dorsal cortex was cut parallel to the longitudinal axis. Cyanoacrylate glue was then used to fix the brain, ventral side up, to an aluminum block. The block was secured at a 12° angle in a Vibratome (Series 1000, Technical Products International, St. Louis, MO), so that the caudal end of the brain faced the blade. Brain slices (400 um) were obtained and incubated in normal ACSF at room temperature for a minimum of 1 h before recording. Normal ACSF contained (mM): 120 NaCl, 2.5 KCl, 2 $CaCl_2$, 2 $MgCl_2$, 25 $NaHCO_3$, and 10 d-glucose, pH 7.4, and was continuously bubbled with 95% O_2 +5% CO_2. For Aβ challenged groups, slices were incubated in 200 nM Aβ for 1 hour. For Cu(II)+Vc treated group, slices were incubated with 200 nM Aβ+Cu(II)+Vc solution.

2) Extracellular recordings: After 1-hour incubation, slices were moved to a recording chamber for extracellular field potential recording. Since cell viability has to be guaranteed through the time course of long-time recording, we have improved the viability of the slice to more than 10 hours by implementing a newly designed *in vitro* slice recording membrane chamber (Hill MR, Greenfield SA, 2011) into our recording system. Slices were perfused with the ACSF during all experiments at room temperature. Schaffer collateral commissural projections were stimulated with a bipolar tungsten electrode (enamel-insulated nichrome wire, 125 mm diameter) for orthodromic activation of the CA1 neurons. A recording borosilicate glass electrode, filled with 150 mM NaCl, was placed in the stratum radiatum (dendritic region) of the CA1 region to record field excitatory postsynaptic potentials (fEPSPs). The recording electrode was placed within 150 mm of the stimulating electrode. Constant current pulses of 0.1 ms duration (15s interval) were generated by a Grass S88 stimulator (Grass Instruments, Quincy, MA) and delivered through an isolation unit. Pulse polarity was selected to produce the largest potentials with a clearly distinguishable artifact. Before each experiment, a stimulus-response (input-output, I/O)

relationship was established to determine the required stimulus giving 60% of the maximum. This control stimulus amplitude was then used for subsequent pair-pulse facilitation (PPF) experiments in that particular slice. For PPF experiments, two pulses of 50 ms intervals were delivered every 15 seconds. PPF ratio was calculated by dividing the magnitude of the second responses by that of the first response. Responses were filtered, amplified, and recorded with a PClamp 10 amplifier (Axon Instruments, Foster city, CA) and acquisition of data was performed using Clampex 10 (Axon Instruments).

3) Drug preparation: The Aβ42 was dissolved in DMSO, oligomerized in PBS, and then incubated for 24 hours at 37° C. To test the effects of Aβ42, slices were cut using the vibratome, identically to the control group, but were incubated in an oxygenated interface holding chamber filled with 163 nM solution of Aβ42 in aCSF (contains (in mM): 120 NaCl, 2.5 KCl, 2 $CaCl_2$, 2 $MgCl_2$, 25 $NaHCO_3$, and 10 D-glucose, pH 7.4). The interface holding chamber was placed in a water bath at 37°C in order to mimic physiological temperature just as in the control. Slices were incubated for at least one hour prior to electrophysiological experiments and each subsequent slice was incubated for 30 min. longer. fEPSPs and PPF were recorded for each slice in aCSF while in the recording chamber. The treatment (CuSO$_4$ and Vitamin C) was incubated with the Aβ42 in PBS in a ratio of 1:10:1 $CuSO_4$/Vitamin C/ Aβ42 for 24 hours at 37° C. The Aβ42 concentration was identical to that in the challenge. Slices were again cut with the vibratome and incubated in an oxygenated interface holding chamber filled with Aβ42, $CuSO_4$, VitaminC, in aCSF. After incubating for at least one hour, the slices were transferred to the recording chamber and perfused with aCSF while IO curves and PPF were recorded.

4) Statistics: Throughout the text, mean ± standard error (SE) was reported. fEPSP amplitudes were given by pClamp 9 software by measuring the maximum negative deflection from baseline. All data was verified for normal distribution and homogeneity of variance. Statistical significance was determined with one way repeated ANOVA followed by the post hoc Bonferroni test, using SigmaStat software (v. 3.0, Aspire Software International, Ashburn, VA). Effects were considered statistically significant at $p < 0.05$.

SI Table 1 The EICR data of fragments from incubation of Aβ42+Cu(II)/Vc in 50% and 100% $H_2{}^{18}O$ buffer

Peptide Sequence	Aβ ¹⁸O_50% Cu/Control	Aβ ¹⁸O_100% Cu/Control	Aβ-¹⁸O 100%/50%
AEDVGSNKGAI	1.131345631	0.828288981	0.732127264
AEDVGSNKGAIIGLMVGGVVIA	1.031942363	0.925247826	0.896608046
AEFRHDSGYE	9.862541244	10.09156666	1.023221745
AEFRHDSGYEV	2.142878347	9.261899608	4.322177049
AIIGLMV	1.123129707	1.253201039	1.115811496
DAEFRH	3.716216678	24.22933751	6.519893646
DAEFRHD	7.079645484	29.00306393	4.096683089
DAEFRHDSGY	2.703282273	7.741994964	2.863923994
DAEFRHDSGYE	3.363975284	10.90835741	3.242698441
DAEFRHDSGYEV	2.77806045	7.258925989	2.612947457
DAEFRHDSGYEVH	2.946037143	14.85168514	5.041241647
DAEFRHDSGYEVHH	1.905621369	7.901606073	4.146472221
DAEFRHDSGYEVHHQ	1.943370431	13.41337428	6.902119155
DAEFRHDSGYEVHHQKL	12.36492082	3.819349266	0.308885865
DAEFRHDSGYEVHHQKLVFFAE	1.680481102	1.609950125	0.958029295
DAEFRHDSGYEVHHQKLVFFAED	1.025510507	1.107082907	1.079543212
DAEFRHDSGYEVHHQKLVFFAEDVG	1.049700444	1.185054701	1.128945603
DAEFRHDSGYEVHHQKLVFFAEDVGS	1.348241012	1.191654344	0.883858549
DAEFRHDSGYEVHHQKLVFFAEDVGSN	1.110986657	0.961997412	0.865894658
DAEFRHDSGYEVHHQKLVFFAEDVGSNK	1.53015048	0.859290676	0.561572661
DAEFRHDSGYEVHHQKLVFFAEDVGSNKGAIIG	1.168039951	0.786006833	0.672928039
DAEFRHDSGYEVHHQKLVFFAEDVGSNKGAIIGL	1.106696808	1.248482102	1.128115753
DAEFRHDSGYEVHHQKLVFFAEDVGSNKGAIIGLM	2.9430894	1.5429	0.524245033
DAEFRHDSGYEVHHQKLVFFAEDVGSNKGAIIGLMV	1.604914366	0.669814288	0.417352042
DAEFRHDSGYEVHHQKLVFFAEDVGSNKGAIIGLMVGGVVIA	1.000354179	1.020587794	1.020226452
EDVGSNKGAI	1.078662117	0.997239244	0.924514942
EDVGSNKGAIIGLMVG	1.106179784	1.360179872	1.229619174
EDVGSNKGAIIGLMVGGVVIA	1.072236642	2.043174305	1.905525539
FAEDVGSNK	1.351811097	1.756693876	1.299511359
FAEDVGSNKG	1.072662429	1.020206613	0.951097555
FAEDVGSNKGA	1.53173717	1.312004087	0.856546484
FAEDVGSNKGAI	1.026155844	0.988640355	0.963440748
FAEDVGSNKGAII	1.026093062	0.976371577	0.951542909
FAEDVGSNKGAIIGL	1.032667065	1.148020265	1.111704153
FAEDVGSNKGAIIGLM	1.013013561	1.183661005	1.168455242
FAEDVGSNKGAIIGLMVGGV	1.610454487	2.916265349	1.810833757
FFAEDV	1.424255162	1.495387862	1.04994379
FFAEDVG	1.456199985	1.303901177	0.895413535
FFAEDVGS	5.476879887	3.357352315	0.613004554
FFAEDVGSNK	1.623008277	1.749099078	1.077689561

FFAEDVGSNKGAI	1.09964354	1.186115092	1.078635985
FFAEDVGSNKGAII	1.019232314	1.029676457	1.010247068
FFAEDVGSNKGAIIG	1.001959328	0.952600397	0.95073759
GLMVGGVVI	1.022138174	1.080204812	1.056808991
GLMVGGVVIA	1.06043447	1.0545053	0.994408735
HDSGYEV	17.94674261	111.2302173	6.197794202
HDSGYEVHHQKLVFFAEDVGSNKGAIIG	3.28313269	2.050993771	0.624706329
KGAIIGLMVGGVVIA	1.024153873	3.102666715	3.029492732
KLVFFAEDVG	3.438583066	0.387808789	0.112781568
LMVGGVVIA	1.411781967	1.290770679	0.914284719
LVFFAEDVGSNKGAIIG	1.768702061	1.908697795	1.079151677
LVFFAEDVGSNKGAIIGL	1.109302699	1.037489824	0.935263049
QKLVFFAEDVGSNKG	1.184693516	1.156548903	0.976243128
QKLVFFAEDVGSNKGAI	1.20245718	0.912916124	0.759208842
QKLVFFAEDVGSNKGAIIG	1.036528655	1.085413192	1.047161781
QKLVFFAEDVGSNKGAIIGLM	2.304877022	1.369931859	0.594362235
VFFAEDVGSNKG	2.790293332	1.26096871	0.451912599
VFFAEDVGSNKGAIIGLMV	1.339824786	1.210360683	0.903372363

Supplemental Figures

The following Nano-LC, MS and MS/MS spectra belong to SI Fig.1

Note: 1. The order of Nano-LC spectra is: Aβ42+Cu(II)/Vc in 0%, 50%, and 100% $H_2{}^{18}O$ buffer

2. The order of MS spectra is: Aβ42+Cu(II)/Vc in 0%, 50%, and 100% $H_2{}^{18}O$ buffer

AEFRHDSGYEV: 437.1984 (light); 437.8665 (heavy)

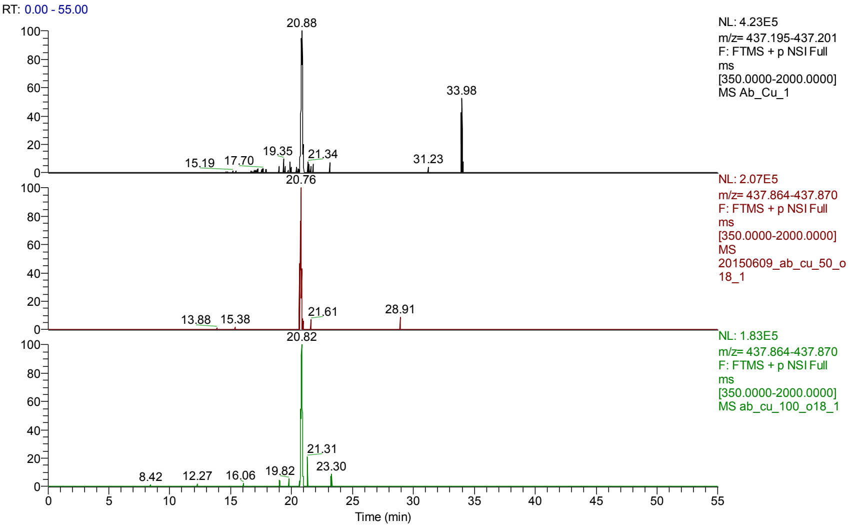

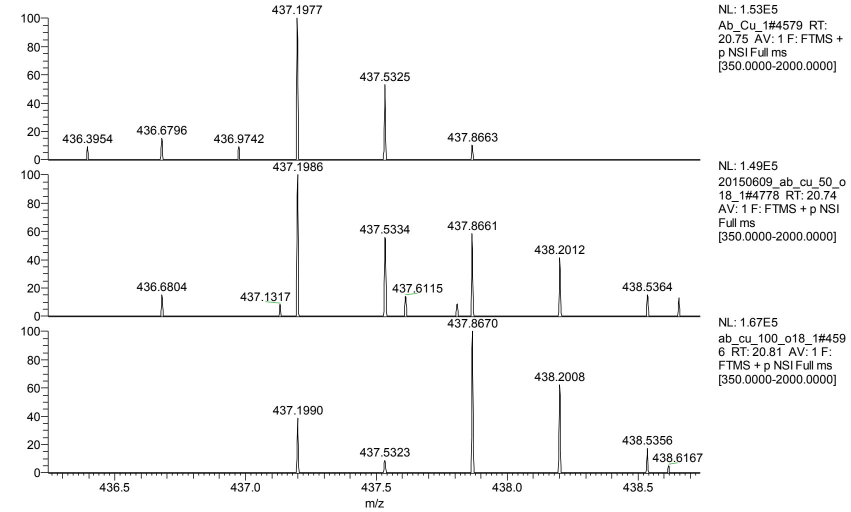

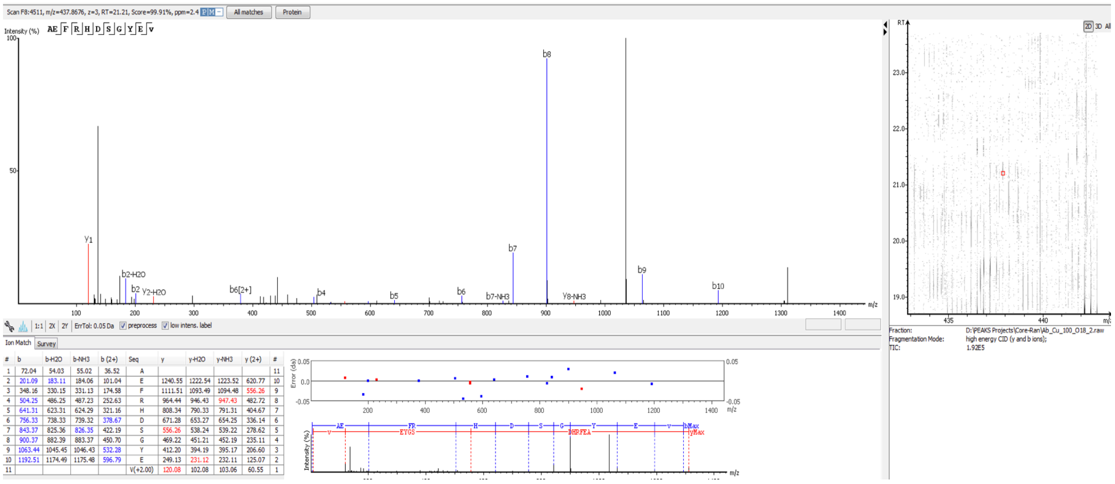

DAEFRH: 387.6801(light); 388.6822 (heavy)

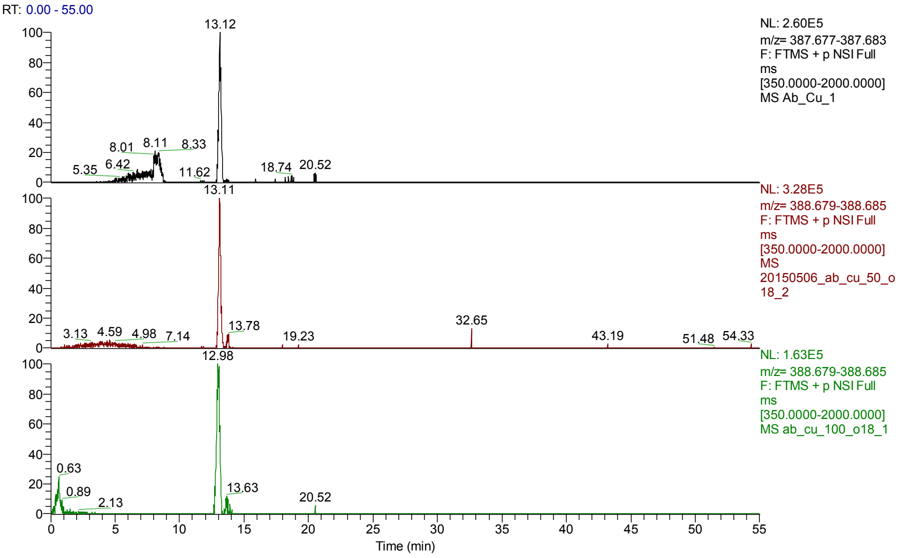

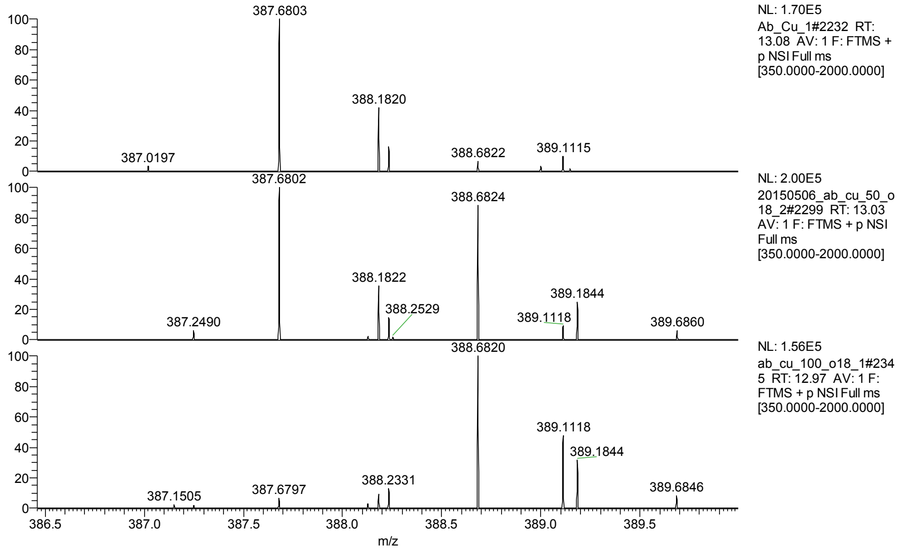

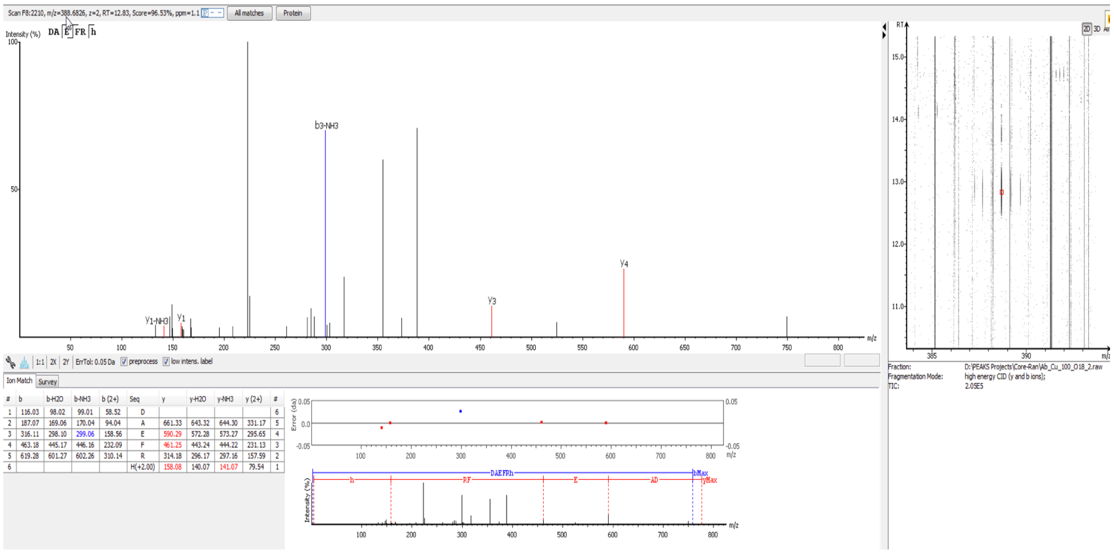

DAEFRHD 445.1936 (light), 446.1957 (heavy)

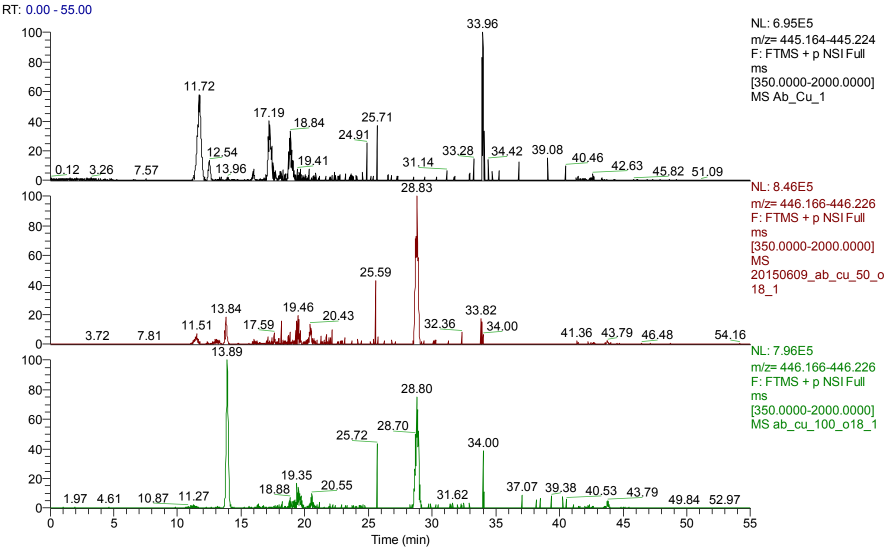

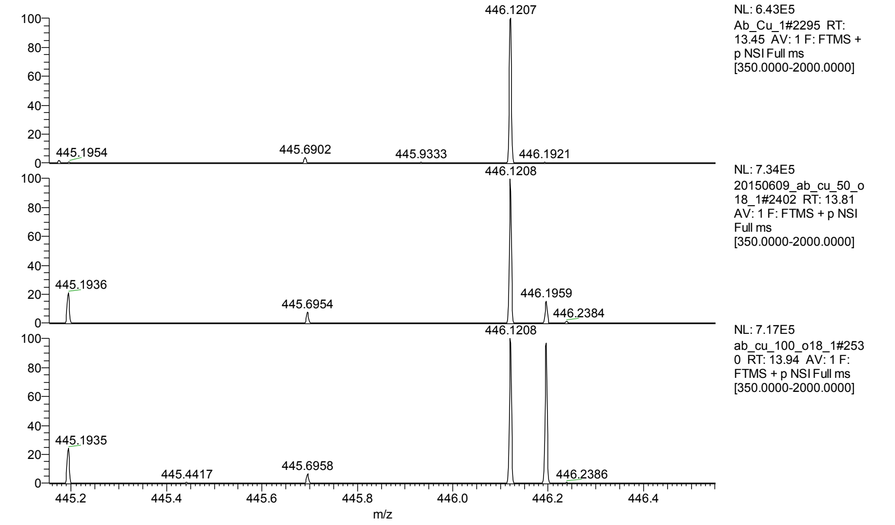

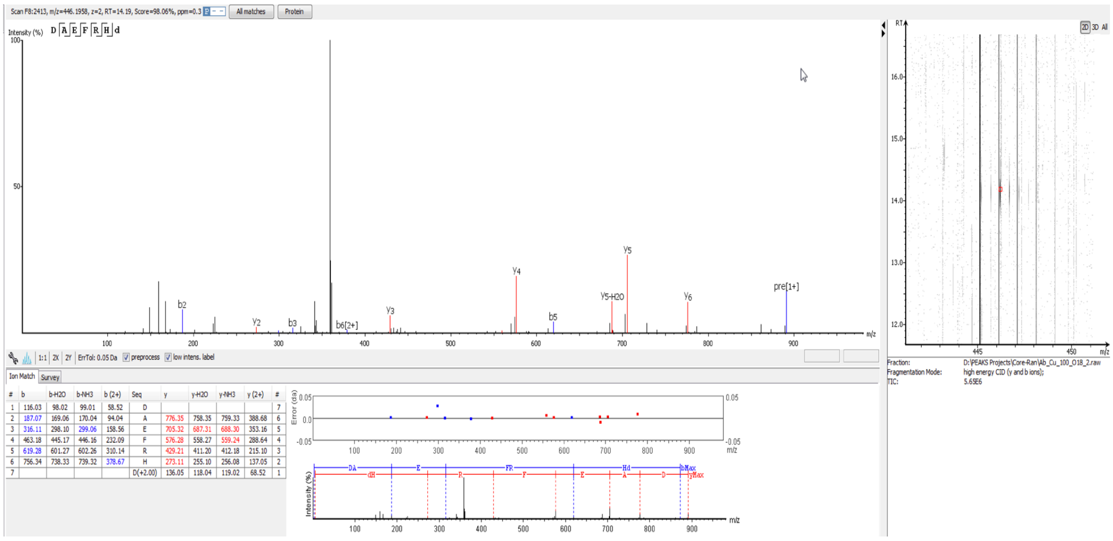

DAEFRHDSGY: 598.7520 (light), 599.7541(heavy)

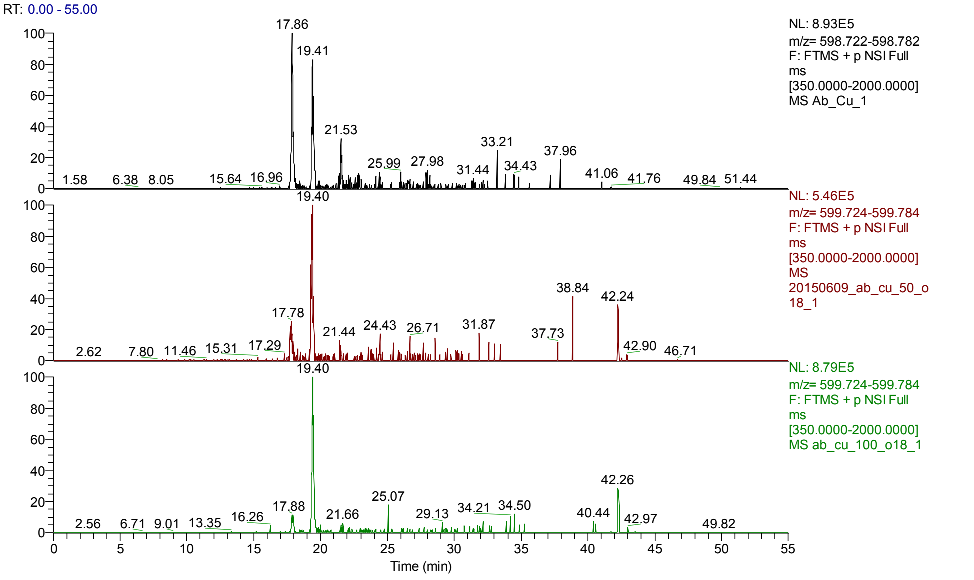

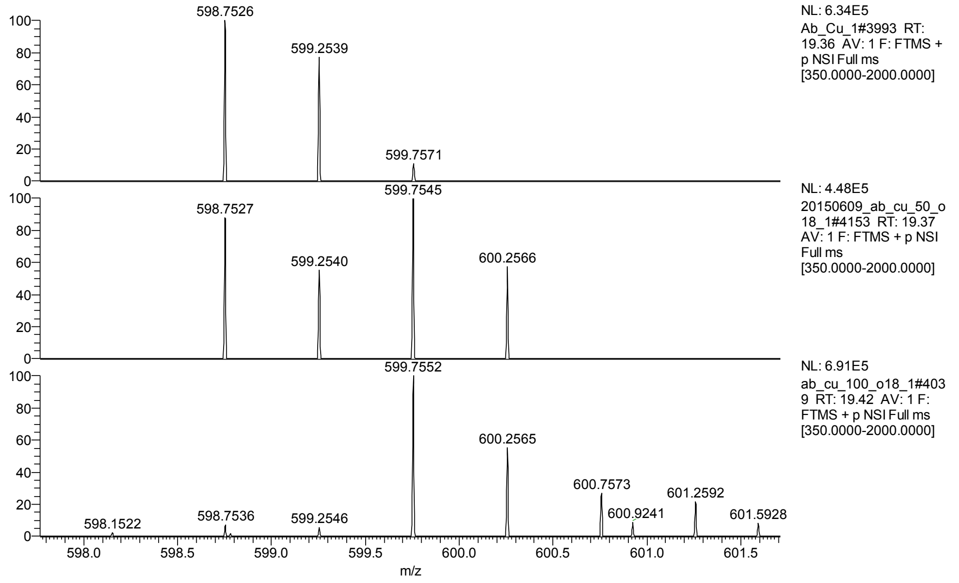

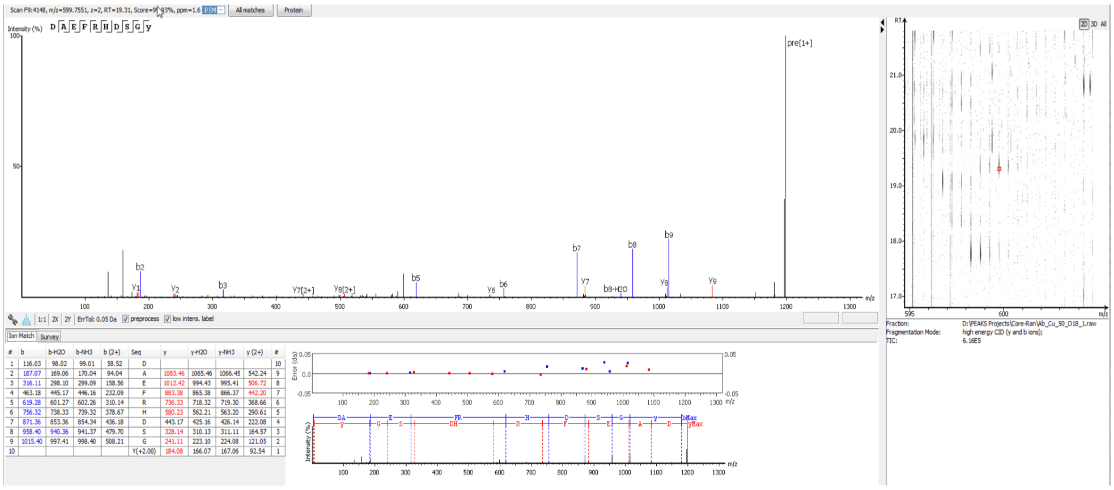

DAEFRHDSGYEV: 475.5408 (light); 476.2088 (heavy)

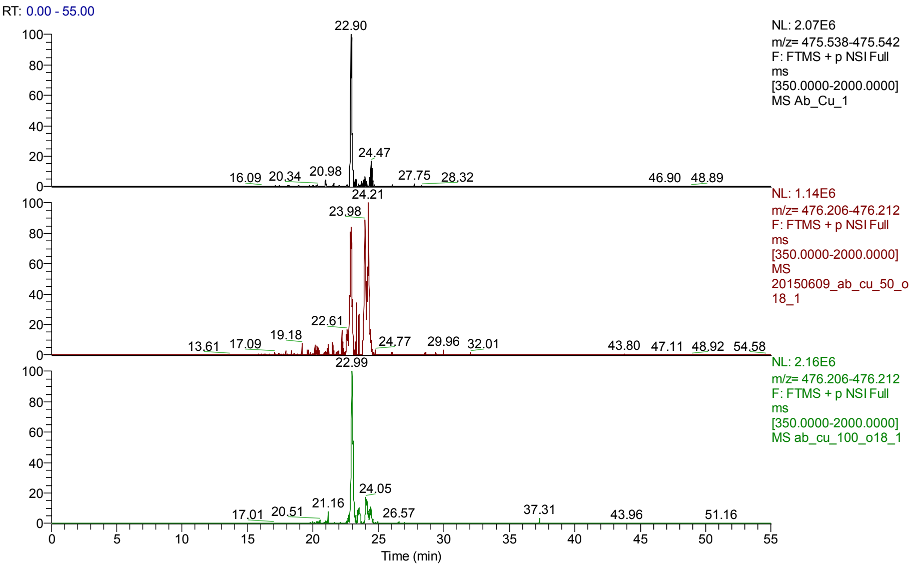

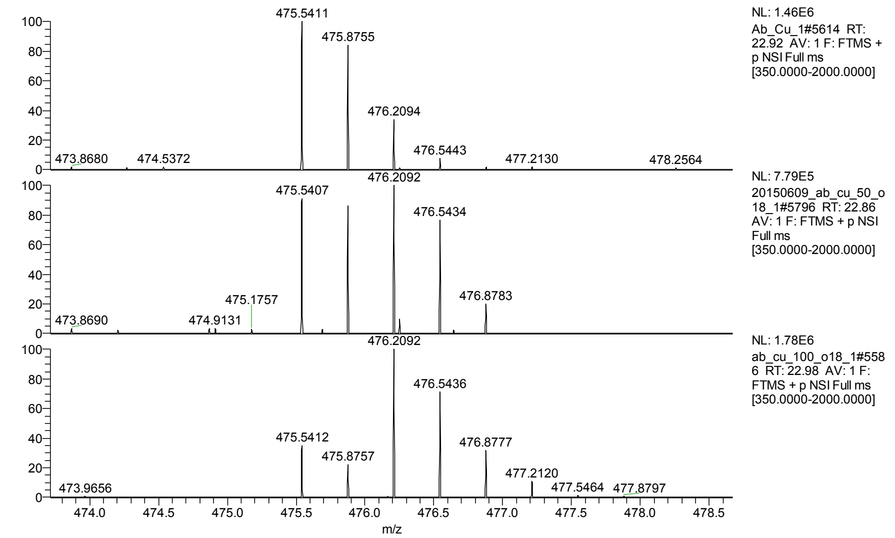

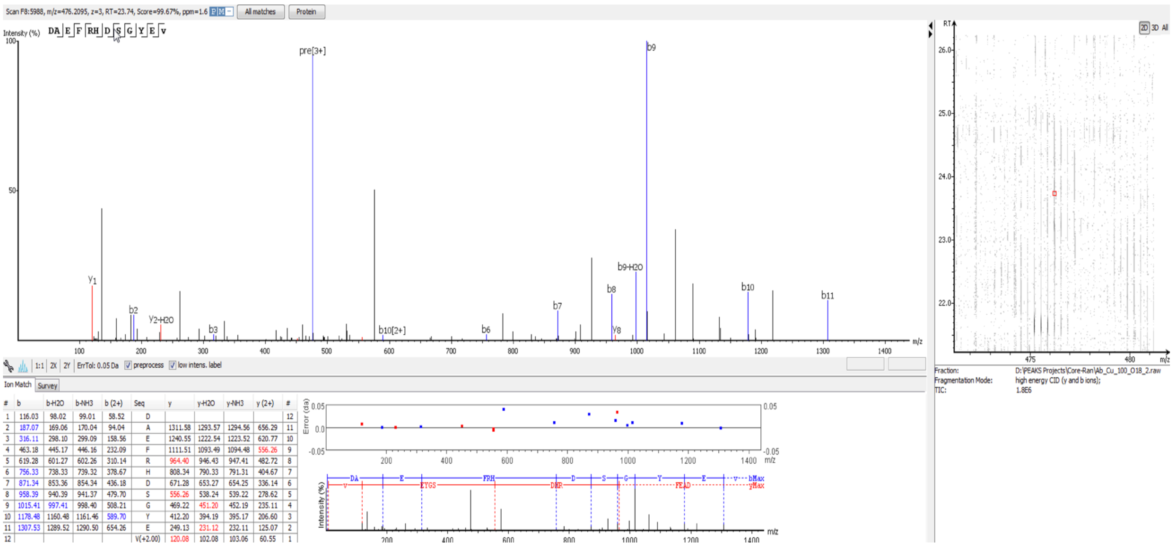

DAEFRHDSGYEVH: 521.2271 (light), 521.8951 (heavy)

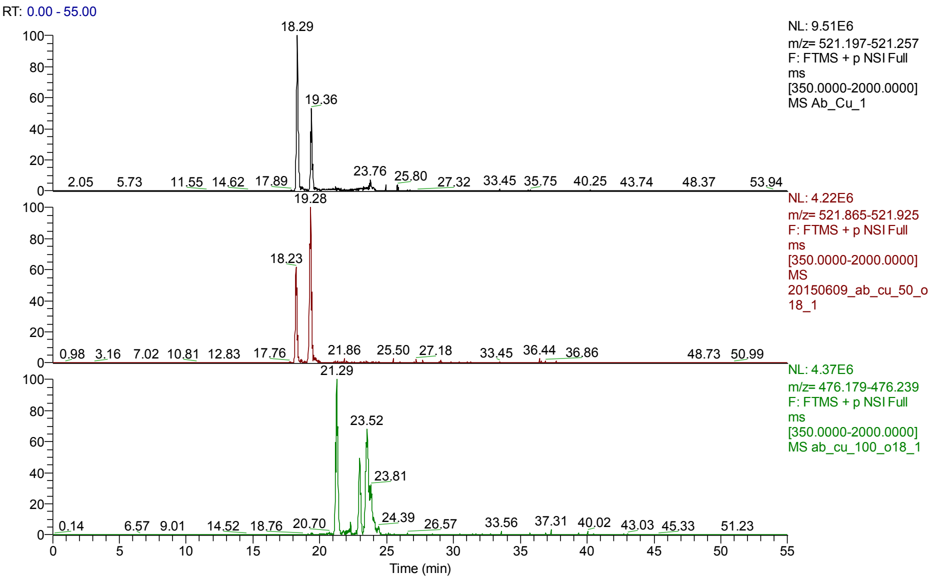

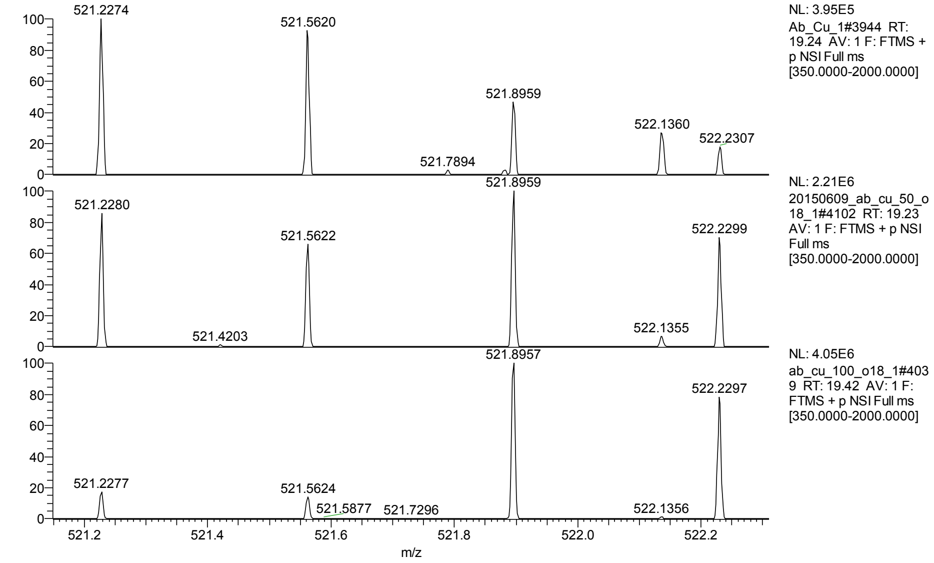

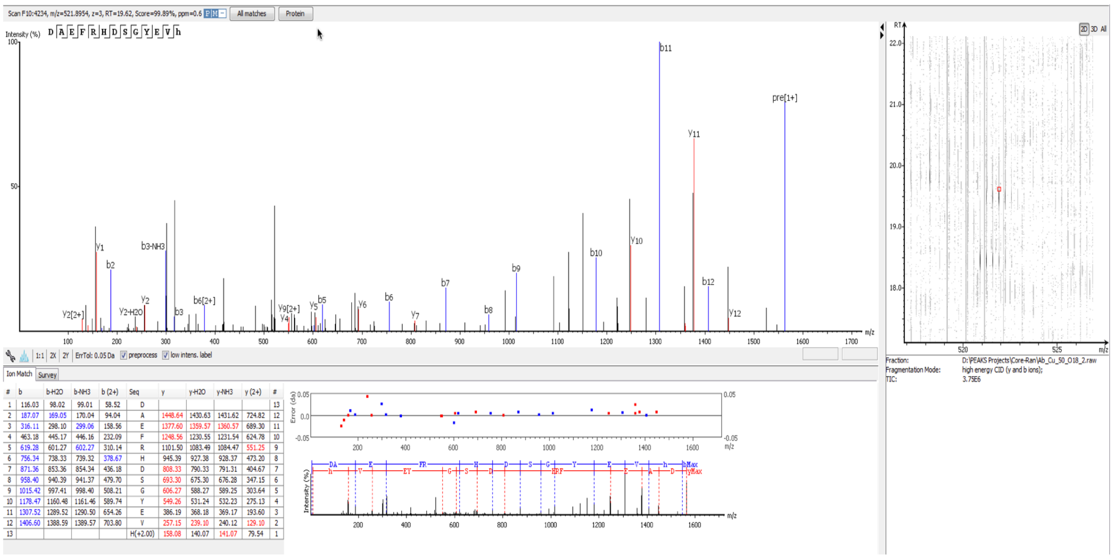

DAEFRHDSGYEVHH: 425.4369 (light), 425.9379 (heavy)

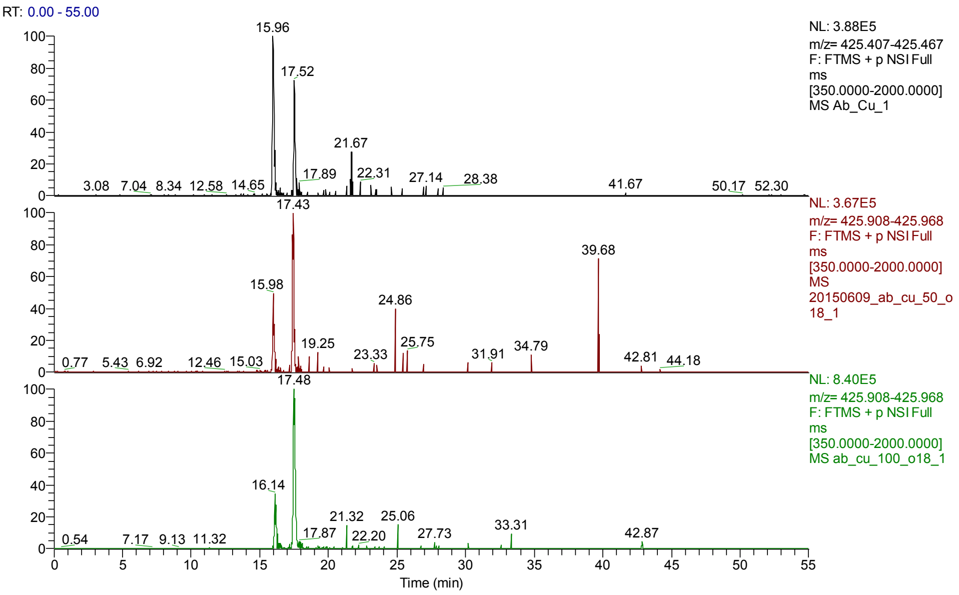

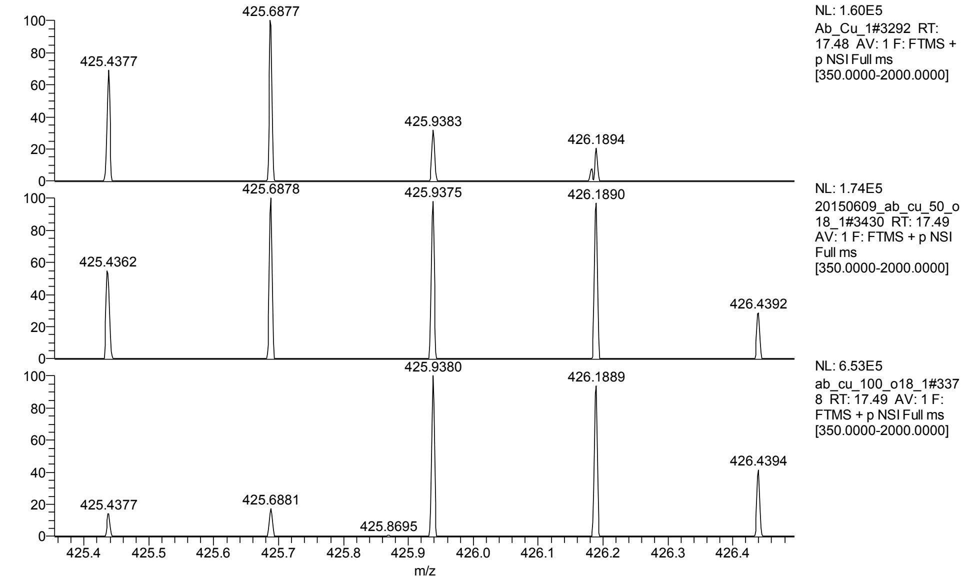

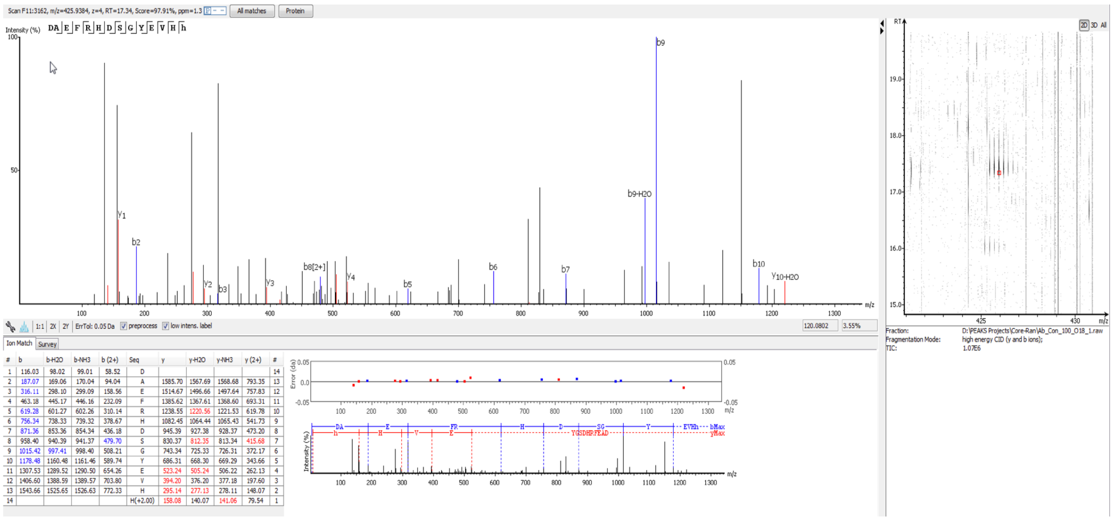

DAEFRHDSGYEVHHQ: 457.4515 (light), 457.9525 (heavy)

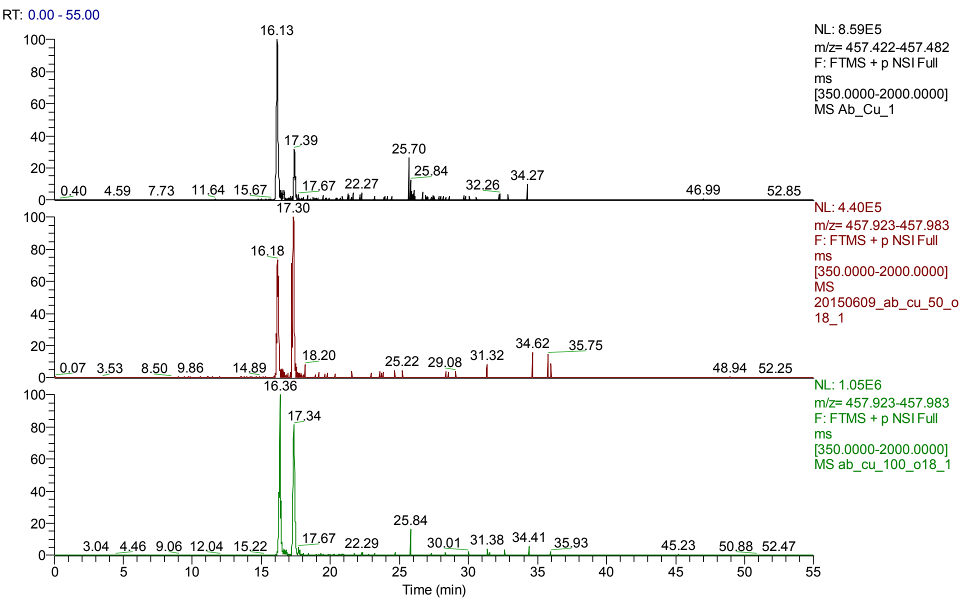

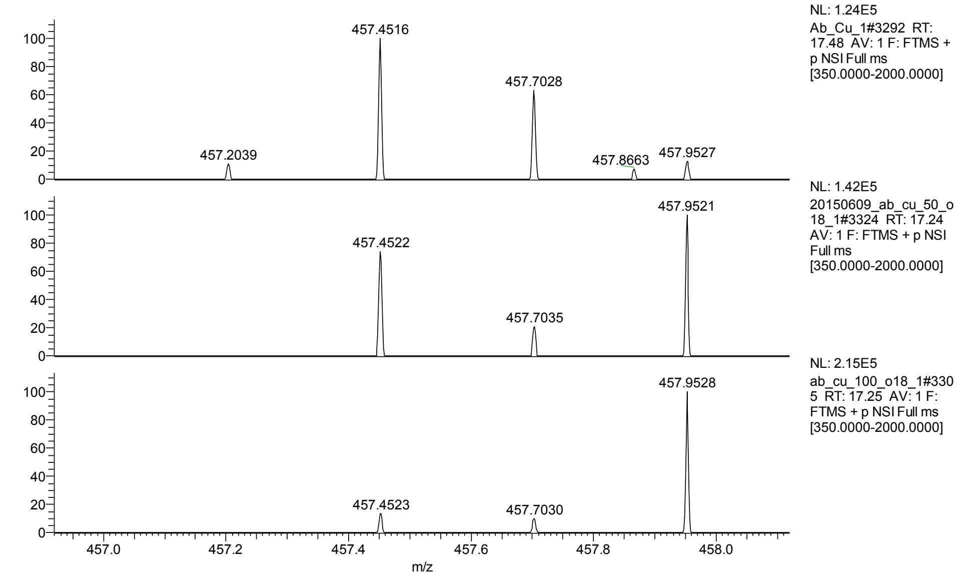

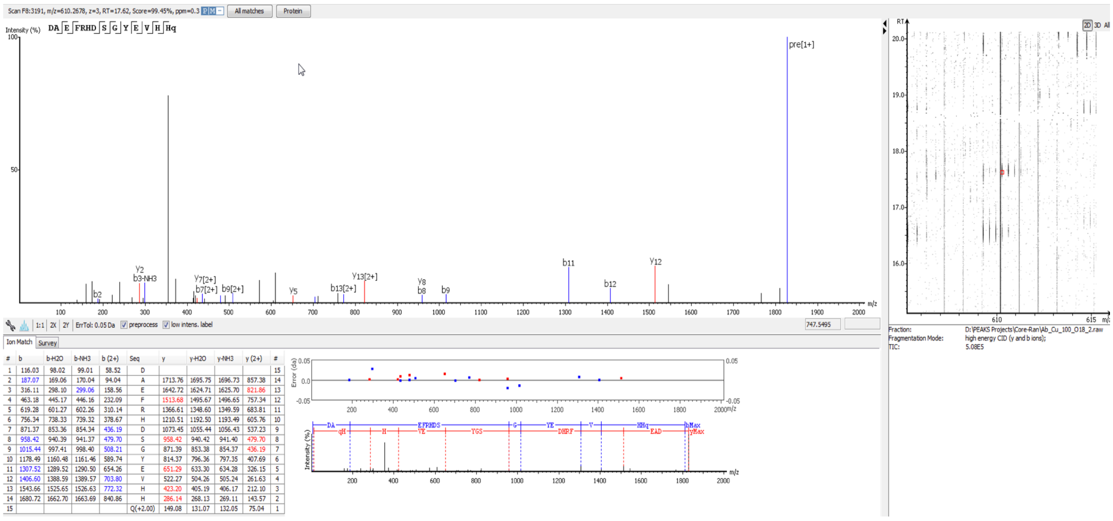

HDSGYEV: 403.6694 (light); 404.6715 (heavy)

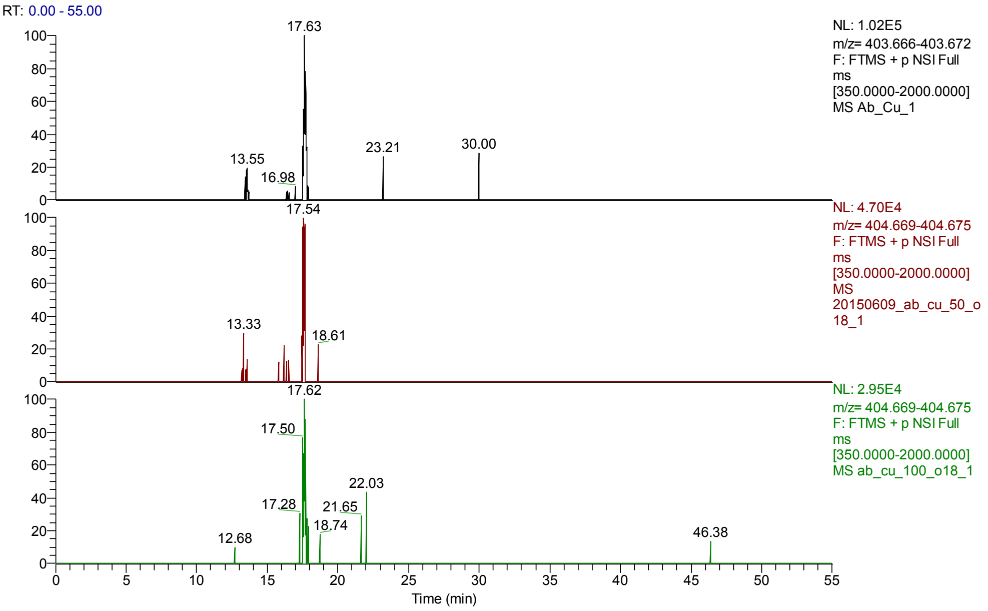

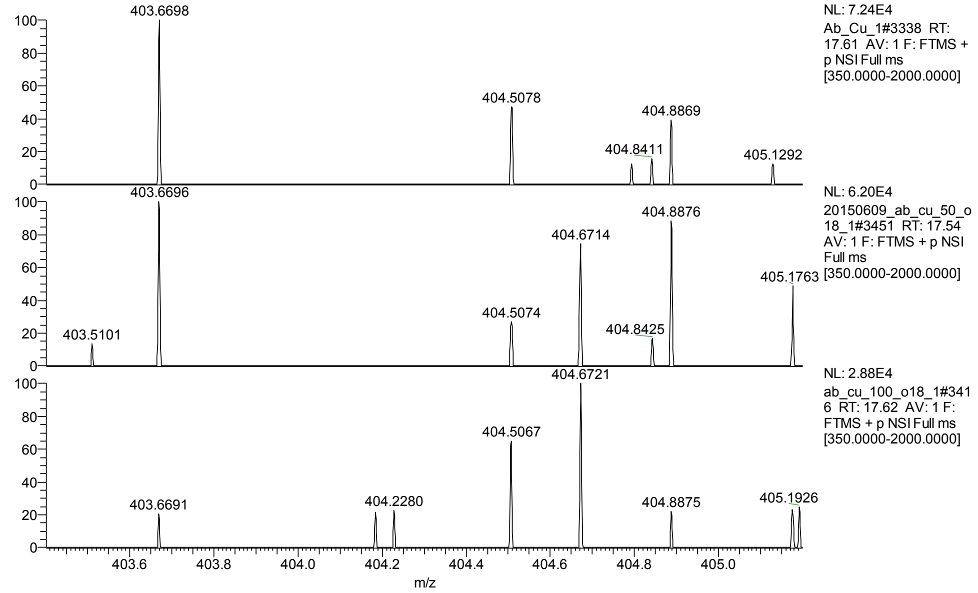

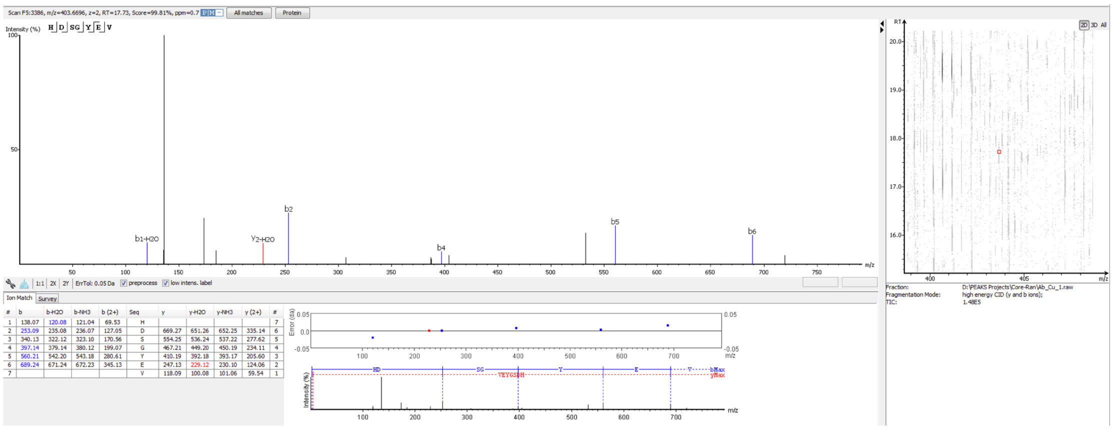

The following Nano-LC, MS, and MS/MS are from the oxidized fragments of Aβ

DAEFRH(oxidized):396.6797 (light), 397.6818 (heavy)

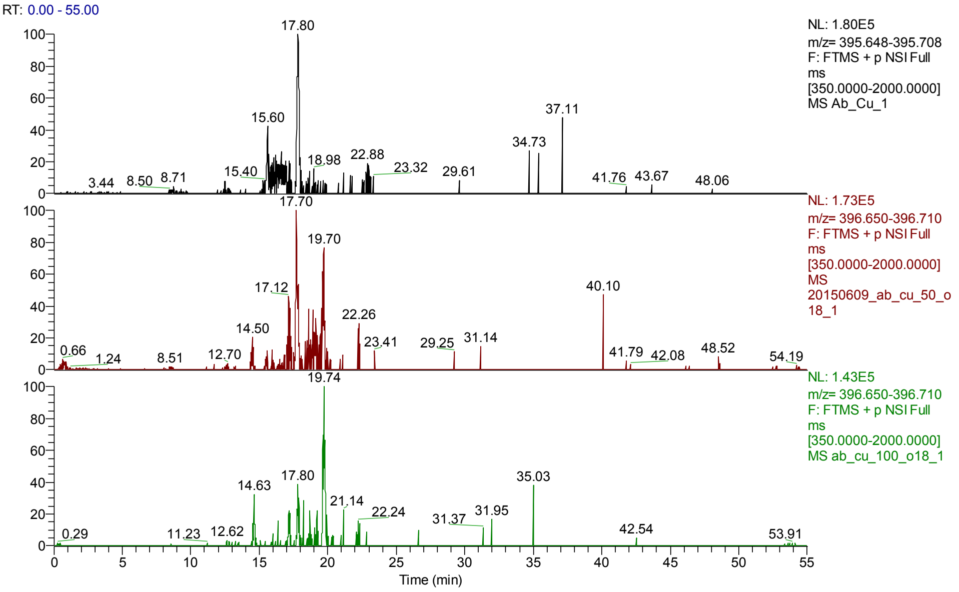

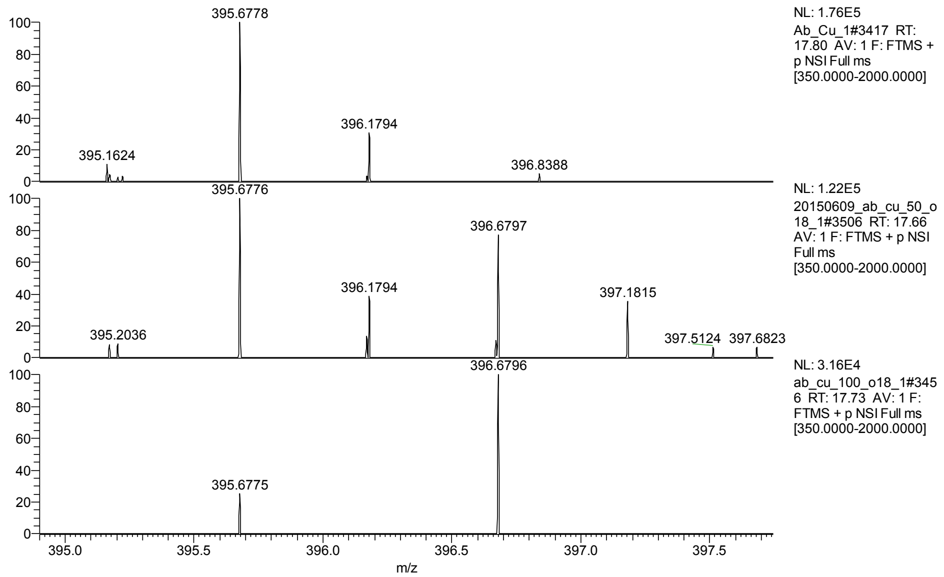

DAEFRH(oxidized)D: 453.1910 (light), 454.1932 (heavy)

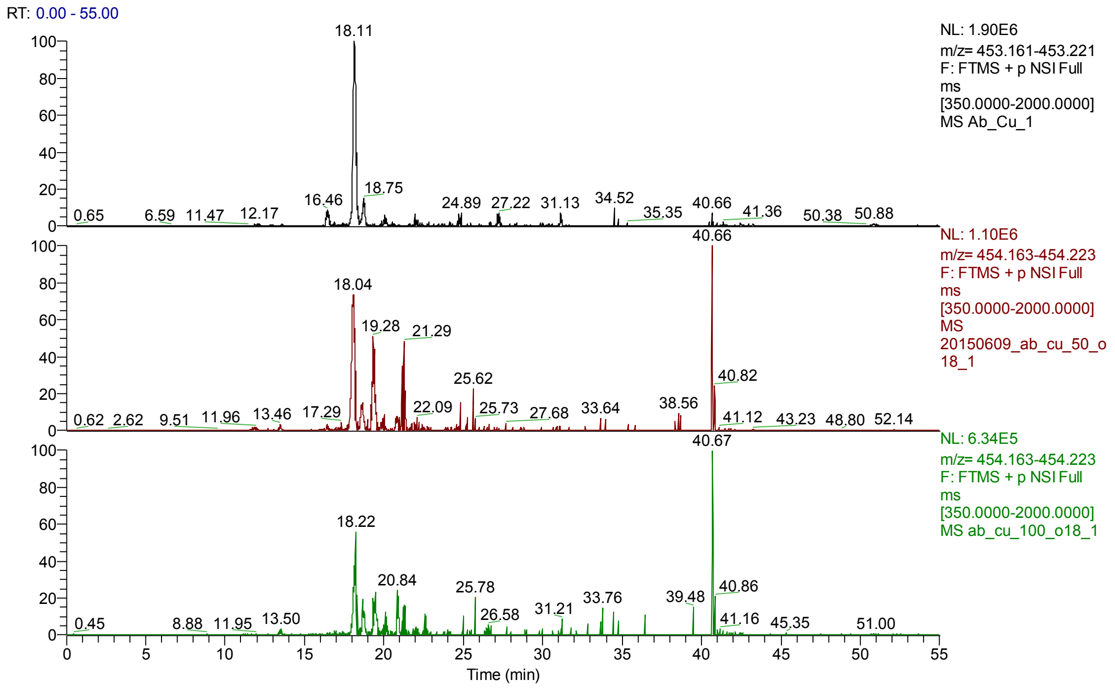

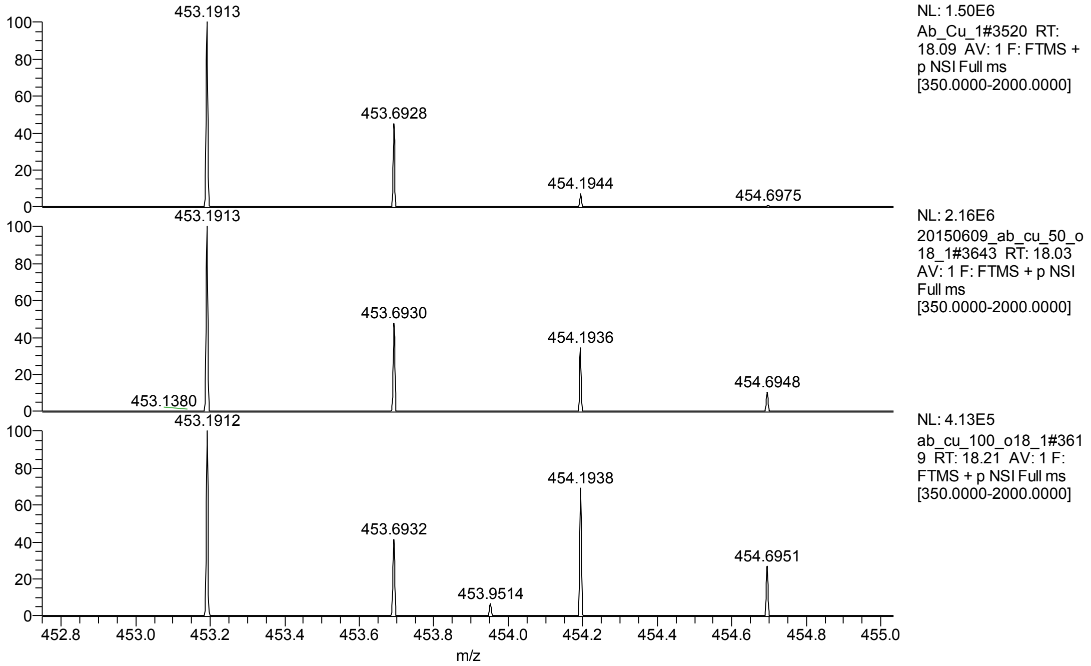

DAEFRH(oxidized)DSGYEV: 720.8049 (light), 721.8071 (heavy)

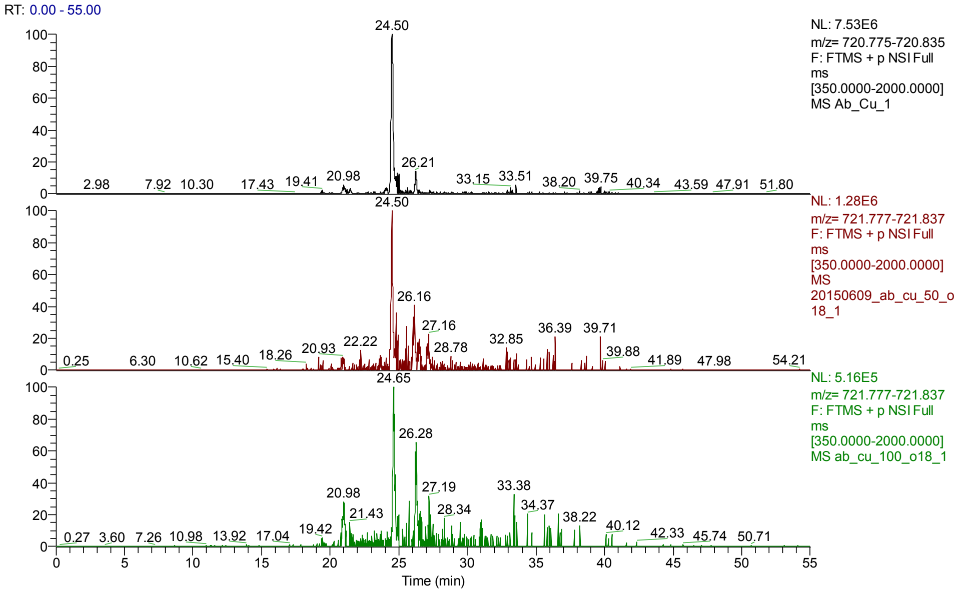
RT: 0.00 - 55.00
NL: 7.53E6
m/z= 720.775-720.835
F: FTMS + p NSI Full ms [350.0000-2000.0000] MS Ab_Cu_1
NL: 1.28E6
m/z= 721.777-721.837
F: FTMS + p NSI Full ms [350.0000-2000.0000] MS 20150609_ab_cu_50_o18_1
NL: 5.16E5
m/z= 721.777-721.837
F: FTMS + p NSI Full ms [350.0000-2000.0000] MS ab_cu_100_o18_1
Time (min)

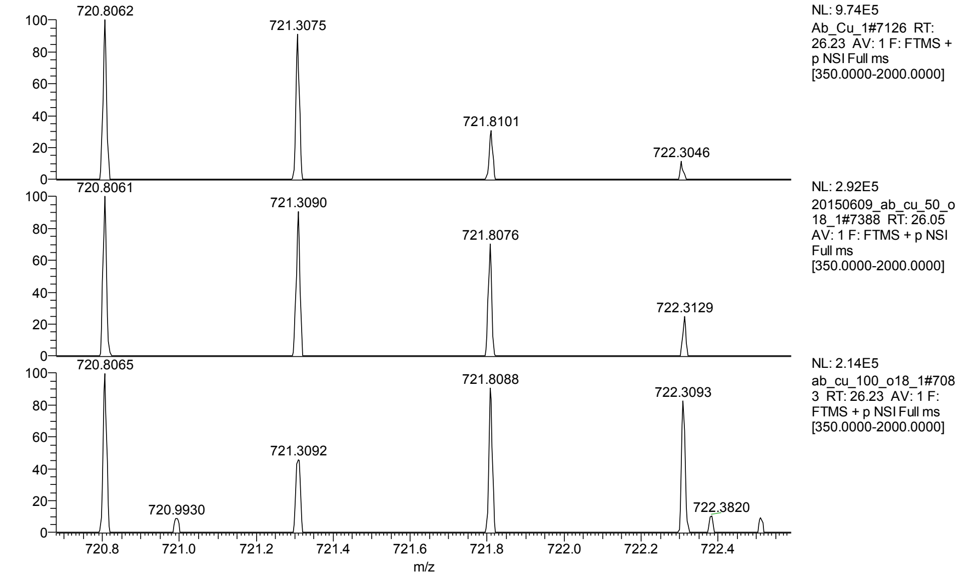
NL: 9.74E5
Ab_Cu_1#7126 RT: 26.23 AV: 1 F: FTMS + p NSI Full ms [350.0000-2000.0000]
NL: 2.92E5
20150609_ab_cu_50_o18_1#7388 RT: 26.05 AV: 1 F: FTMS + p NSI Full ms [350.0000-2000.0000]
NL: 2.14E5
ab_cu_100_o18_1#7083 RT: 26.23 AV: 1 F: FTMS + p NSI Full ms [350.0000-2000.0000]
m/z

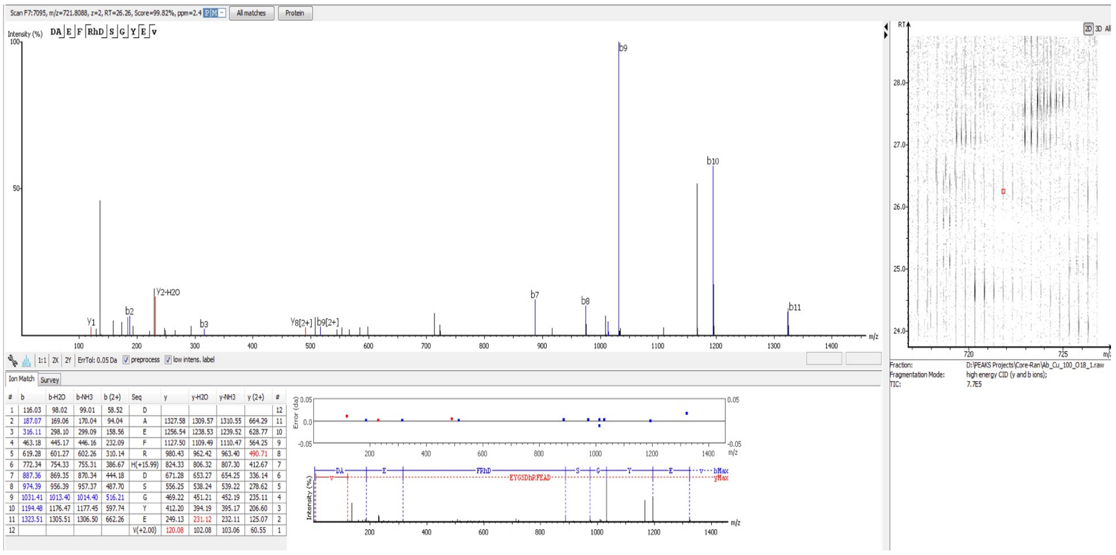

DAEFRH(oxidized)DSGYEVH: 526.5587 (light), 527.2268 (heavy)

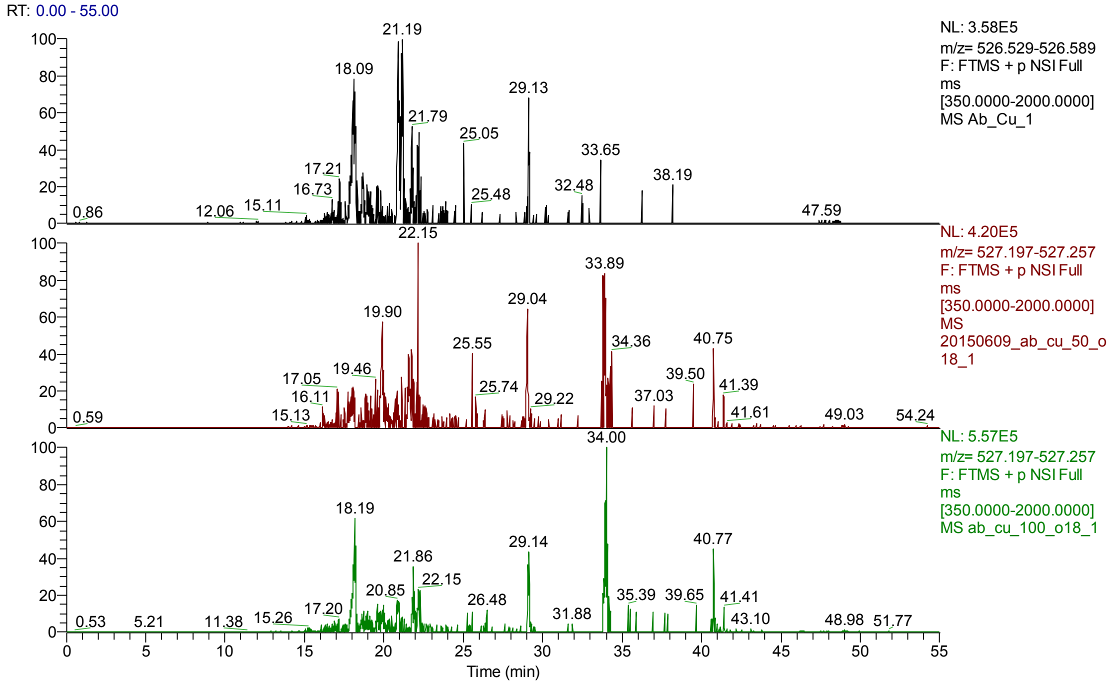

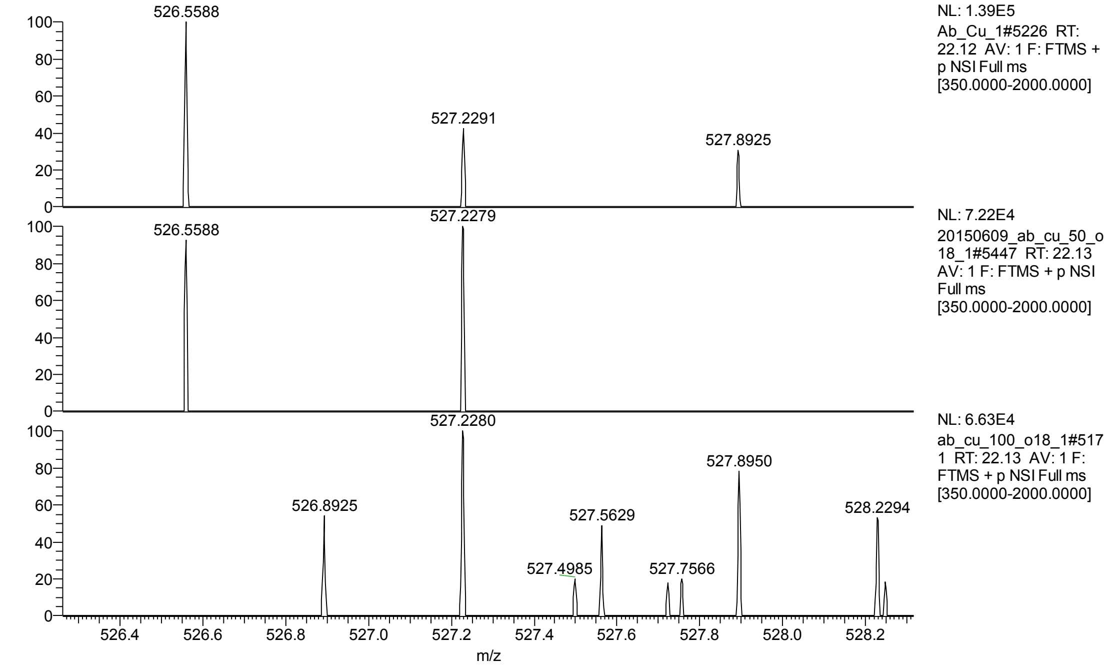

The following Nano-LC, MS and MS/MS spectra belong to SI Fig.2

Note: 1. The order of Nano-LC spectra is: Aβ42+Cu(II)/Vc in 0%, 50%, and 100% $H_2^{18}O$ buffer

2. The order of MS spectra is: Aβ42+Cu(II)/Vc in 0%, 50%, and 100% H_2^{18} Obuffer

FAM-DAEFRH: 378.1384; 378.8065 (heavy)

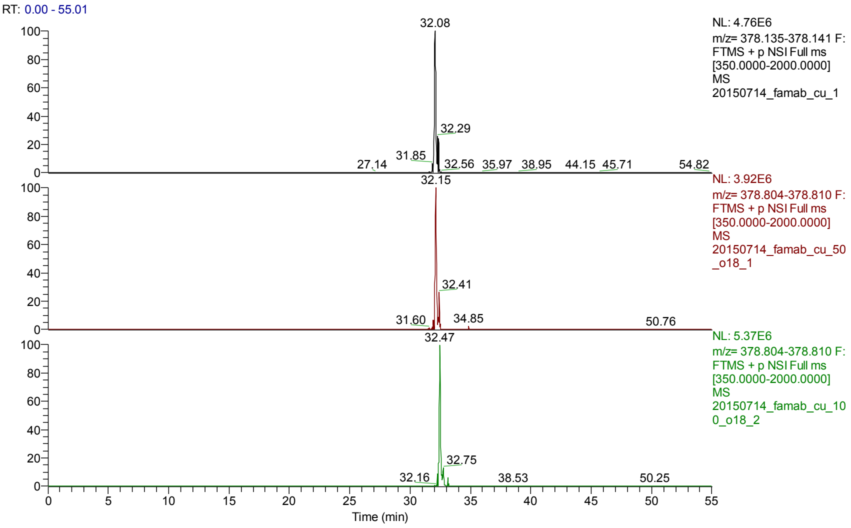

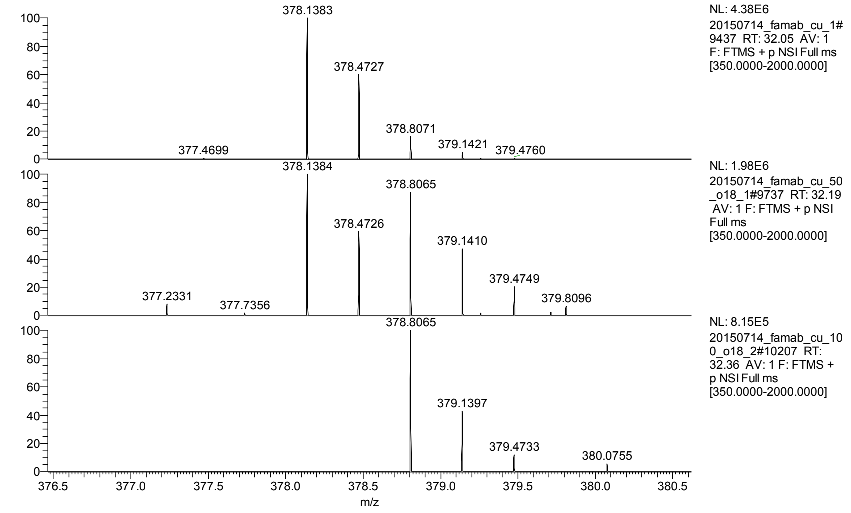

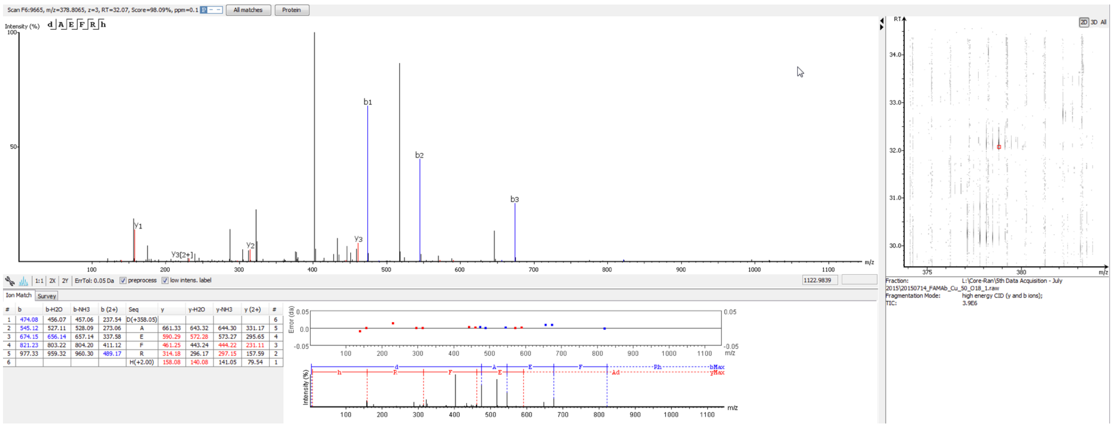

FAM-DAEFRHDSGYEV: 594.8900; 595.5581 (heavy)

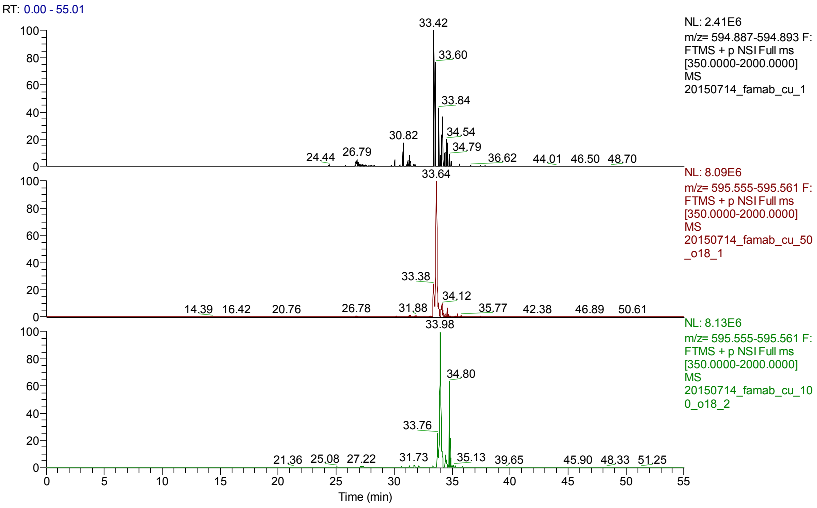

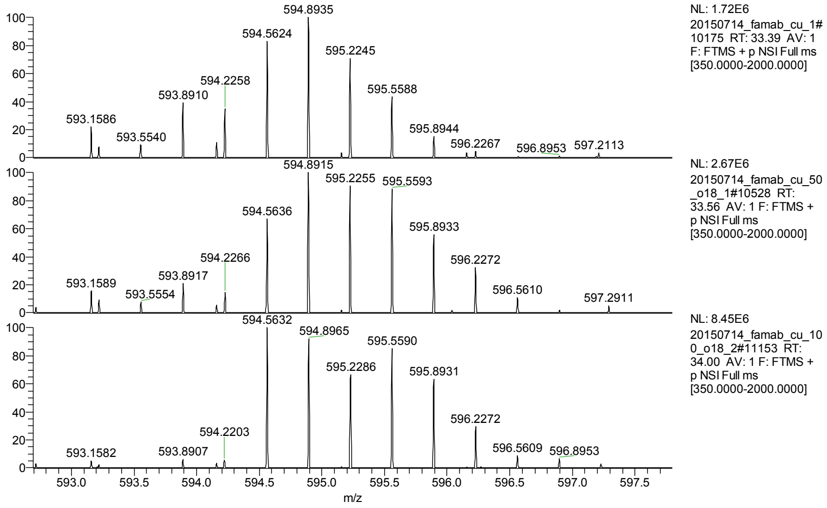

FAM-DAEFRH(oxidized)DSGYEV: 899.8288; 900.8309 (heavy)

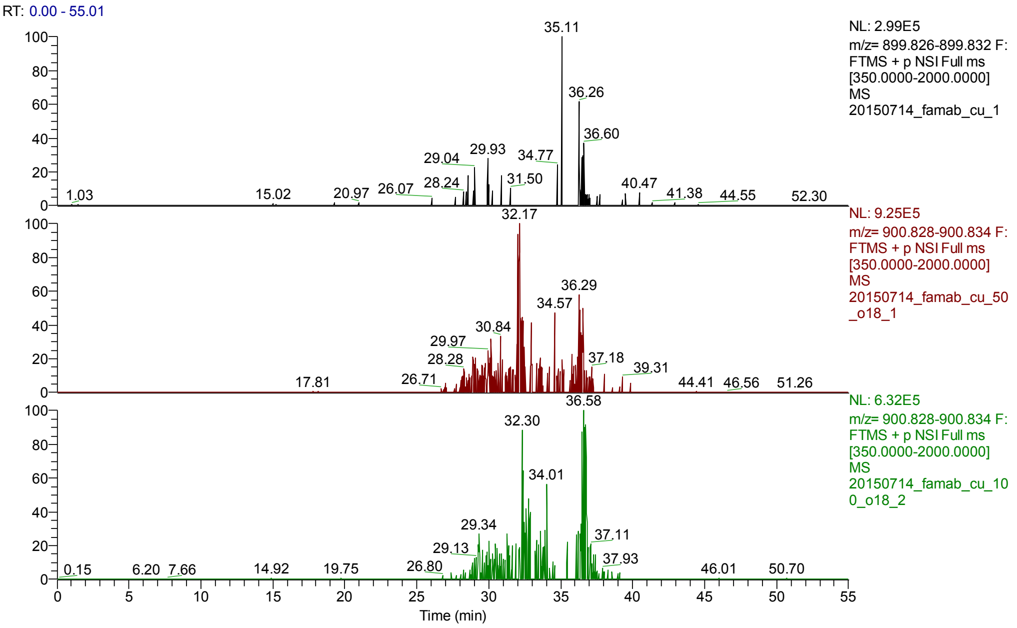

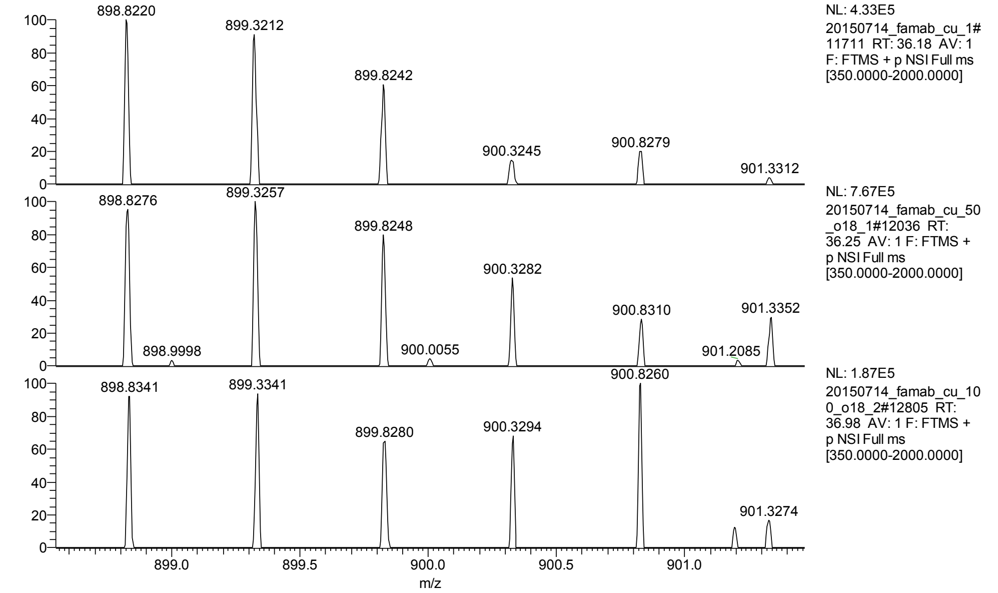

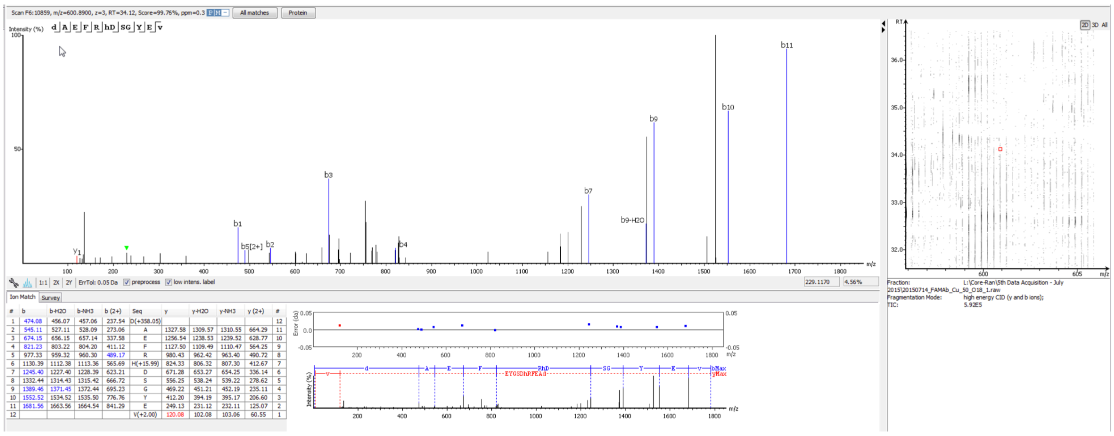

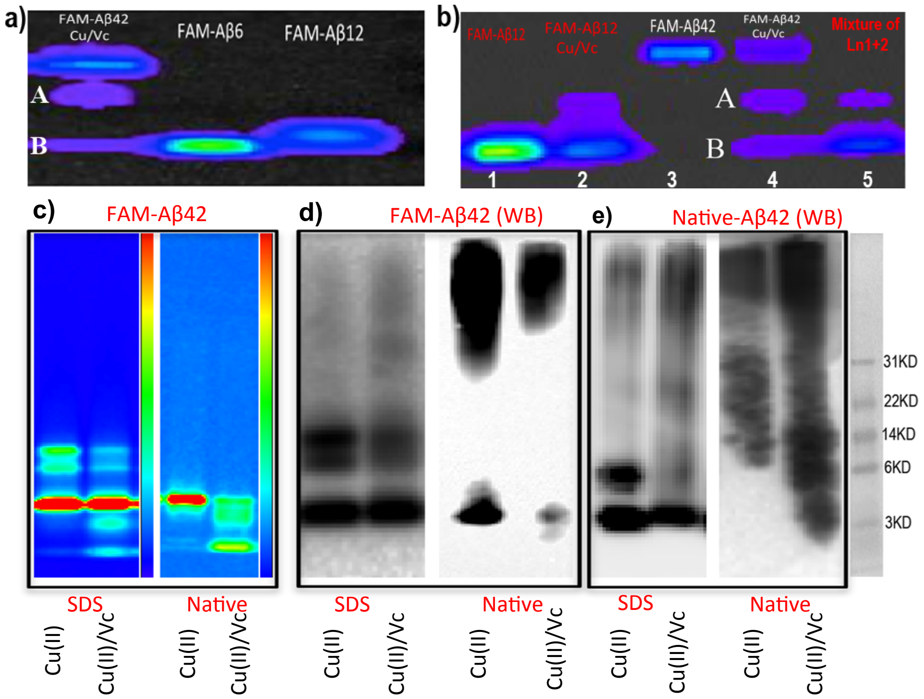

SI Fig.3 a) SDS-Page gel of FAM-Aβ42 with Cu(II)/Vc, and standard FAM-Aβ6 and FAM-Aβ12. It is clear that the positions of band B and FAM-Aβ6 are similar. b) SDS-Page gel of FAM-Aβ12, and FAM-Aβ12 with Cu(II)/Vc, standard FAM-Aβ42, FAM-Aβ42 with Cu(II)/Vc, and mixture of lane 1 and lane2. It is clear that the positions of band A and the oxidized FAM-Aβ12 (lane 2) are similar. c) Fluorescence images of FAM-Aβ42 with Cu(II) and Cu(II)/Vc from SDS-gel and native gel. d) Western blots of FAM-Aβ42 with Cu(II) and Cu(II)/Vc from SDS-gel and native gel. e) Western blots of native-Aβ42 with Cu(II) and Cu(II)/Vc from SDS-gel and native gel.

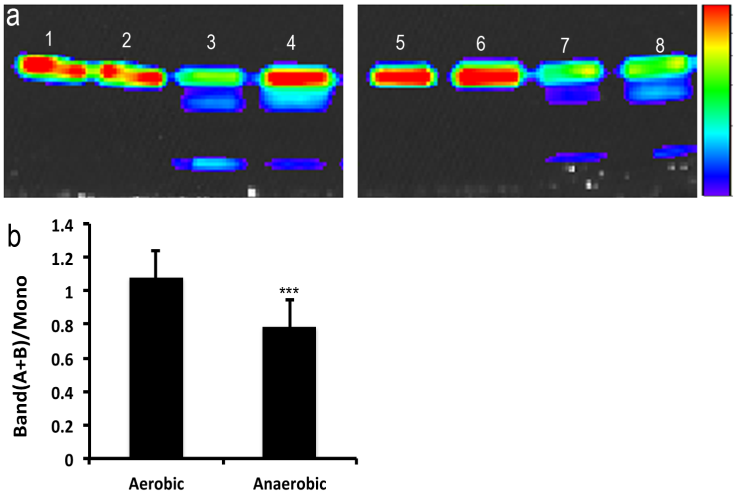

SI Fig.4 (a) SDS-PAGE of FAM-Aβ42 with Cu(II) under both aerobic and anaerobic conditions with and without Vc and H_2O_2. **Lane 1-4, aerobic condition:** FAM-Aβ42 (Lane1), FAM-Aβ42/ Cu(II) (Lane2), FAM-Aβ42/ Cu(II) /Vc (Lane3), FAM-Aβ42/Cu(II)/V_C/H_2O_2 (Lane 4); **Lane 5-9, anaerobic condition:** FAM-Aβ42 (Lane 5); FAM-Aβ42/Cu(II) (Lane 6); FAM-Aβ42/Cu(II)/Vc (Lane 7), FAM-Aβ42/ Cu(II) /V_C /H_2O_2 (Lane 8). **(b)** Quantitative analysis of Lane 3 and 7 (n = 4) via ratios of Band (A+B)/Mono.

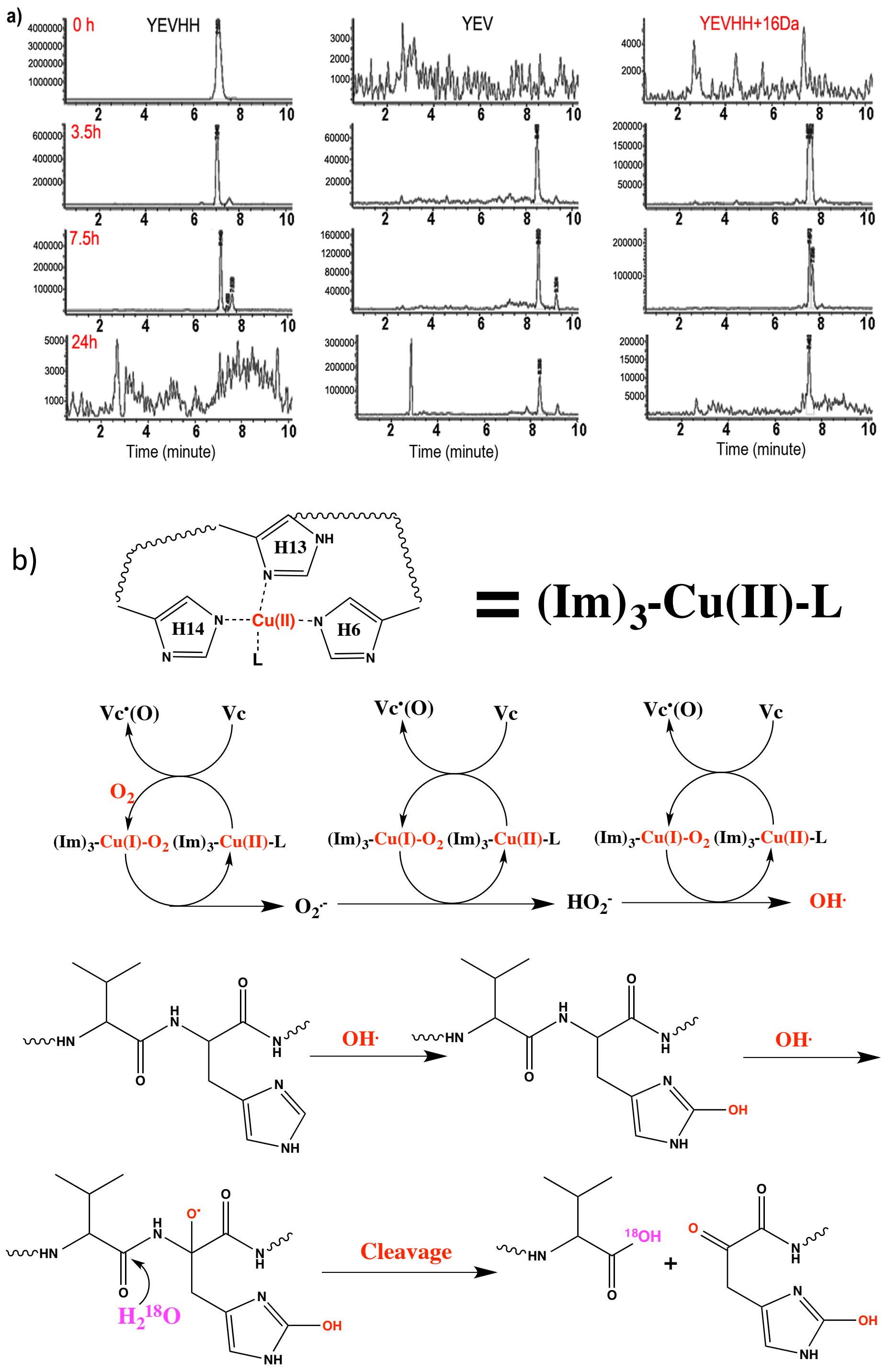

SI Figure 5. Preliminary cleavage mechanism studies. **(a)** LC-MS of a model peptide YEVHH (left panel), the degraded product YEV (middle panel) and the oxidized intermediate YEVHH+16Da (right panel) after 0 h, 3.5h, 7.5h, and 24h incubation. *Note, the scales of Y axis of XIC (extracted ion current) are different for each LC-MS.* **(b)** A tentative mechanism for the copper induced degradation, which is proposed based on the cleavage of the model peptide YEVHH and the degraded fragments of Aβ peptides.

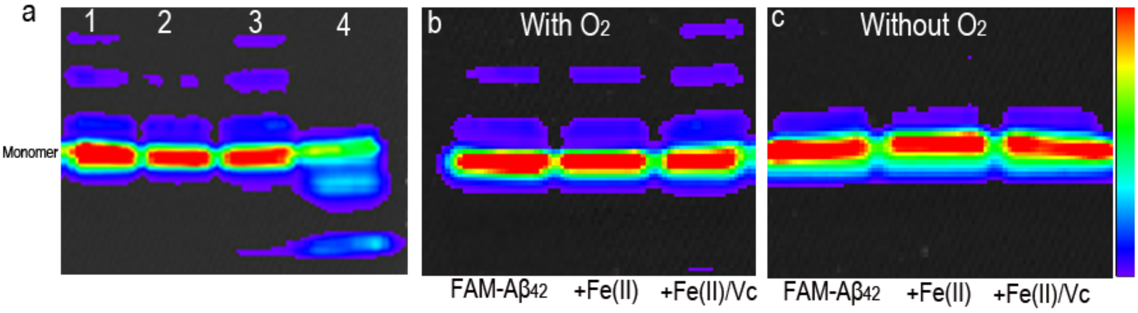

SI Fig.6 a) SDS-PAGE of FAM-Aβ42 with different ratio of Cu(II) and Vc. FAM-Aβ42/ Cu(II) =1:0.1(Lane1), FAM-Aβ42/ Cu(II) /Vc=1:0.1:0.1(Lane 2), FAM-Aβ42/ Cu(II) /Vc=1:0.1:20 (Lane 3), FAM-Aβ42/ Cu(II) / Vc=1:1:20 (Lane 4). b) SDS-PAGE of FAM-Aβ42 with Fe(II) and Fe(II)/Vc under an aerobic condition. c) SDS-PAGE of FAM-Aβ42 with Fe(II) and Fe(II)/Vc under an anaerobic condition. It is clear that no cleaved bands could be observed.

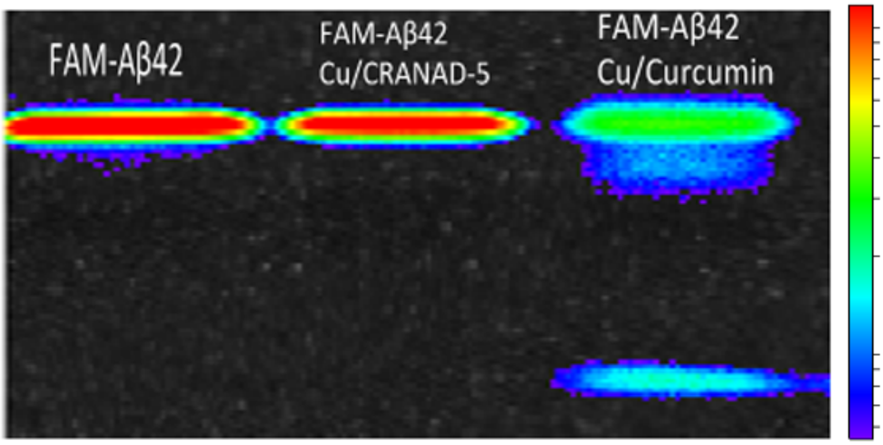

SI Fig.7 SDS-Page gel of FAM-Aβ42 only, FAM-Aβ42 with Cu(II)/CRANAD-5, and FAM-Aβ42 with Cu(II)/curcumin. It is clear that no apparent degraded fragment is observed when FAM-Aβ42 was incubated with Cu(II)/CRANAD-5.

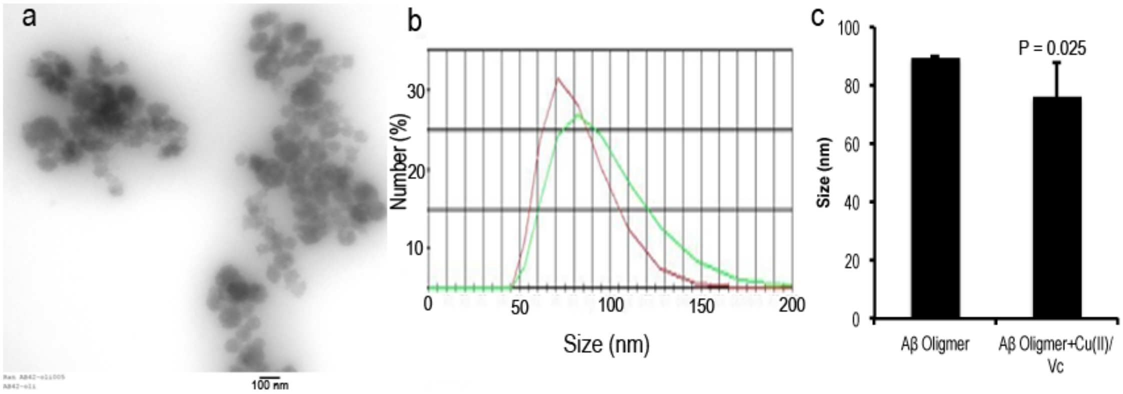

SI Fig 8. A) Representative TEM image of Aβ42 oligomers. b) Size distribution of Aβ42 oligomers with and without Cu(II)/Vc treatment. c) Quantitative analysis of the sizes of Aβ42 oligomers (n=4).

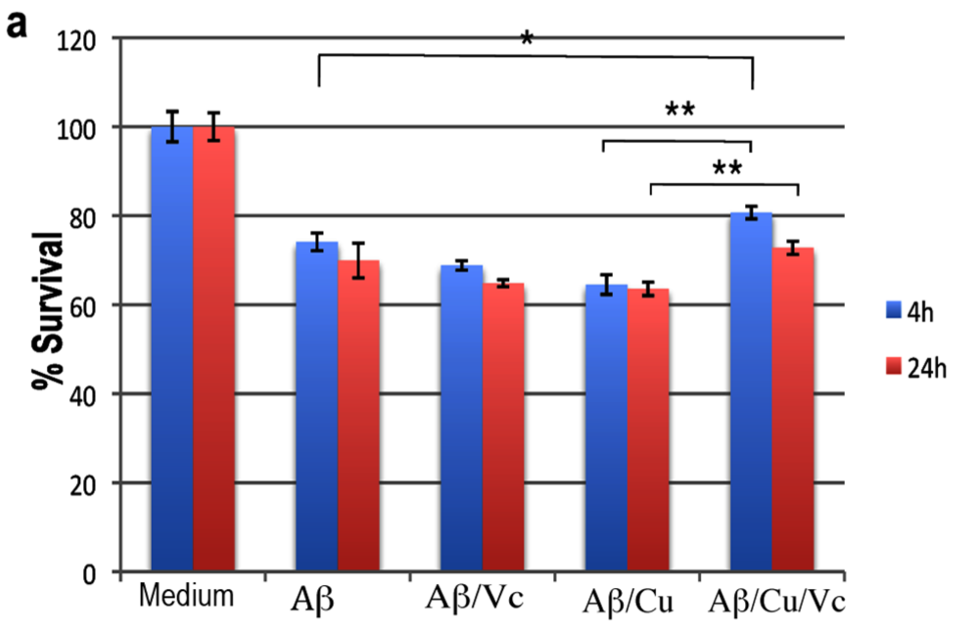

SI Fig. 9 MTT neurotoxicity testing with different treatments.
